# Understanding Emotions: Origins and Roles of the Amygdala

**DOI:** 10.3390/biom11060823

**Published:** 2021-05-31

**Authors:** Goran Šimić, Mladenka Tkalčić, Vana Vukić, Damir Mulc, Ena Španić, Marina Šagud, Francisco E. Olucha-Bordonau, Mario Vukšić, Patrick R. Hof

**Affiliations:** 1 Department of Neuroscience, Croatian Institute for Brain Research, University of Zagreb Medical School, 10000 Zagreb, Croatia; vukic.vana@gmail.com (V.V.); espanic@hiim.hr (E.Š.); mariovuksic@net.hr (M.V.); 2 Department of Psychology, Faculty of Humanities and Social Sciences, University of Rijeka, 51000 Rijeka, Croatia; mlat@ffri.hr; 3 University Psychiatric Hospital Vrapče, 10090 Zagreb, Croatia; damir.mulc@hotmail.com; 4 Department of Psychiatry, Clinical Hospital Center Zagreb and University of Zagreb School of Medicine, 10000 Zagreb, Croatia; marinasagud@mail.com; 5 Department of Medicine, School of Medical Sciences, Universitat Jaume I, 12071 Castellón de la Plana, Spain; folucha@uji.es; 6 Nash Family Department of Neuroscience and Friedman Brain Institute, Icahn School of Medicine at Mount Sinai, New York, NY 07305, USA; patrick.hof@mssm.edu

**Keywords:** amygdala, emotion, evolution, fear, anxiety

## Abstract

Emotions arise from activations of specialized neuronal populations in several parts of the cerebral cortex, notably the anterior cingulate, insula, ventromedial prefrontal, and subcortical structures, such as the amygdala, ventral striatum, putamen, caudate nucleus, and ventral tegmental area. Feelings are conscious, emotional experiences of these activations that contribute to neuronal networks mediating thoughts, language, and behavior, thus enhancing the ability to predict, learn, and reappraise stimuli and situations in the environment based on previous experiences. Contemporary theories of emotion converge around the key role of the amygdala as the central subcortical emotional brain structure that constantly evaluates and integrates a variety of sensory information from the surroundings and assigns them appropriate values of emotional dimensions, such as valence, intensity, and approachability. The amygdala participates in the regulation of autonomic and endocrine functions, decision-making and adaptations of instinctive and motivational behaviors to changes in the environment through implicit associative learning, changes in short- and long-term synaptic plasticity, and activation of the fight-or-flight response via efferent projections from its central nucleus to cortical and subcortical structures.

## 1. Introduction

Emotions played a major role in survival during human evolution and in effective psychological functioning in human societies [1]. Unlike reflexes—automatic and uncontrollable narrowly-tuned responses to specific stimuli—emotions emerged and were selected in evolution because they better addressed problems of adaptation to a constantly changing environment [2]. Among others, adaptive abilities to find food, water and shelter, to find sexual partners (mates), to provide adequate protection, nurturing, and care for offspring, and most importantly, to avoid danger and escape from life-threatening situations were probably critical [3]. It has been speculated that emotions initially arose when reflexes were “decoupled” to include another layer of nerve cells on top of them—the evolutionary emergence of central emotional states [4].

**Figure 1:**
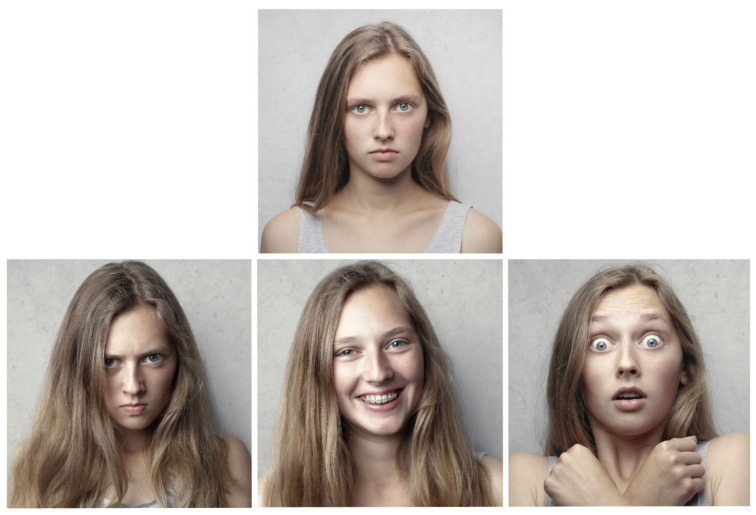
**Emotional facial expressions of three basic, primary emotions.** At the top is a neutral facial expression. In the bottom row, facial expressions of anger, joy, and fear are shown, respectively. Although individual emotions can be recognized and analyzed even from the microexpressions of facial muscles, for the sake of clarity the expressions of emotions in these photographs are accentuated. See text for details. Photographs by Andrea Piacquadio, taken from [9].

Most contemporary theories of emotion are based on the assumption that emotions are biologically determined [3]. Consistent with this biological approach is the finding that some basic, primary emotions, such as anger, fear, joy, sadness, disgust, and surprise, are innate, expressed in the first six months of life, and associated with specific facial expressions. As such, they have been equally recognized in different cultures around the world [5]. According to Ekman and others, different facial expressions of primary emotions are interpreted and reproduced similarly across different cultures [6,7]. Although people in different cultures are relatively equally successful at recognizing facial expressions of basic, primary emotions [5], estimating the intensity of these expressions, however, depends on the cultural context [8]. An illustration of facial expressions of the three primary emotions is shown in Figure 1.

Darwin was probably the first to study the evolution of emotional reactions and facial expressions systematically and to recognize the importance of emotions for the adaptation of the organism to various stimuli and environmental situations [10]. After a detailed description of individual facial expressions as well as the motor apparatus involved in the expression of each individual emotion in his 1872 book, *The expression of emotions in man and animals*, he concluded that emotions in humans, just as in animals, have a common evolutionary history [11]. By presenting the findings that certain emotional facial expressions have universal meaning for people in different parts of the world, Darwin anticipated research of facial expressions that would not begin until more than a century later. From an evolutionary perspective, emotions allow for the coordination of a whole range of different processes with the goal of resolving immediate and urgent issues [12,13,14].

## 2. Classical Theories of Emotion

Some of the first theories of emotion attempted to explain the close relation between physiological changes and the subjective experience of an emotion or a feeling. James, Lange, and Sergi independently assumed, counterintuitively, that subjective emotional experience is caused by changes in the body [15,16,17]. What they meant was that fear, for example, is experienced due to bodily changes brought about by a specific environmental stimulus and that interpretation of that physical response due to changes in the autonomic nervous system (ANS) results in an emotional experience. In their view, after being faced with a frightening stimulus, a physiological response to that stimulus would occur before the subjective experience of an emotion.

James defined in 1884 that “the bodily changes follow directly the perception of the exciting fact, and that our feeling of the same changes as they occur is the emotion” [17]. Specific brain areas (e.g., visual or auditory cortices) process a particular stimulus and evaluate its meaning and relevance. If the stimulus is emotionally important, the information is relayed to the ANS, whose activation leads to a fight-or-flight response. The “conscious part” of the brain then detects bodily arousal and interprets the emotional nature of the experienced physiological state [18]. According to James, different emotions are experienced differently because they arise from different constellations of physiological responses. This James–Lange theory, the first theory of emotion, was later modified and called the peripheral theory of emotions (see below) because it emphasizes the importance of bodily responses for the emergence of emotions [19,20]. One of the examples that speaks in favor of James’s theory is the effect of benzodiazepines, a class of anxiolytic drugs, which are also muscle relaxants [4]. According to the theory, tense muscles signal anxiety to the brain. So, when muscles relax, the brain no longer receives this information and the subject becomes less anxious.

Damasio has recently complemented and reformulated the peripheral theory of emotions [19,20]. His reasoning can be summed up in the claim that emotions are unconsciously formed in the central nervous system (CNS) based on interoceptive and proprioceptive afferent body signals and correlate, to a large extent, with consciously produced feelings in the later course of processing the initial stimuli (this interpretation overlaps with the somatic marker theory [21], see below). Although this theory does not provide a holistic view of emotions and their processing, it has significantly contributed to the idea that emotional experiences involve knowing one’s current and previous bodily states, which is the basis of the concept of embodied cognition [22]. According to Damasio, without the self-representation of one’s own image (of the whole body) and its constant updating, adults would be as helpless as newborns because emotions unaccompanied by conscious feelings would not be sufficient for survival. However, once embodied, emotions can exist exclusively within the CNS, as exemplified by deafferentation phenomena, such as phantom pain. The CNS must consistently update all information about the state of the body to regulate all the processes that keep it alive as the only way an organism can maintain homeostasis and survive in a constantly changing environment. According to the concept of embodied cognition, emotions are grounded throughout the individual as well as its entire personal experience involving the adaptation of all systems to sensory experience [23]. Damasio proposed that the main difference between humans, apes, and other animals is the level and elaboration of body self-image, which in humans, is extremely large (broader core self-image) and includes autobiographical memory, while in other species, it includes only a significantly lower level (core self-image), depending on the degree of cortical development [19,20,24]. Damasio’s proposal also implies that there is no pure perception (i.e., interpretation without bodily experiences) and that by controlling motor behavior and its consequences on proprioception and interoception, one could regulate one’s emotions and thus influence feelings. This concept is used, for example, in dance psychotherapy, where the therapist helps the patient to evoke, process, and regulate certain emotions through movement [25]. Likewise, exploring and practicing new and yet unknown motor patterns can help a person experience new, hitherto unusual feelings [25]. The same principle explains the relatively small but significant finding that the use of botulinum toxin A applied to the muscles used in frowning (mm. corrugatores supercilii) leads to a better mood [26], whereas it leads to a bad mood when applied to the muscles required for laughing (*mm. risorii*, *mm. zygomatici majores*). Consequently, forced laughing leads to a small but significant, greater subjective feeling of contentment and happiness over time (the facial muscle feedback loop, also known as the facial feedback hypothesis) [27,28,29].

Contrary to the James–Lange theory, Cannon and, later, Bard hypothesized that the subjective experience of emotion occurs simultaneously and independently of autonomous bodily changes, which they assumed are always of a similar magnitude no matter what emotion is involved (a view that was refuted by Ekman and others only in 1983) [30]. They also believed that bodily changes are slower than emotions, such that the addition of hormones cannot change the emotional state (now demonstrated not to be true, as an intravenous cholecystokinin injection can cause a panic attack, whereas cortisol, D-cycloserine, and orexin have direct influence on anxiety levels, fear conditioning and extinction) [31,32], as well as that complete surgical separation of the abdominal organs does not change the emotional behavior of animals. Cannon believed that bodily reactions (increased heart rate, glucose mobilization, centralization of the blood circulation, and other effects) were the response of the organism to a sudden, threatening situation, leading to maximal activation of the sympathetic nervous system and preparing the body for the fight-or-flight response [33]. Not accepting James’ hypothesis that “every emotion is tied to a distinct body state”, his interpretation was that all emotional events affecting the sympathetic nervous system lead to general, non-discriminatory physical arousal. Moreover, he believed that the CNS is capable of eliciting any emotion, even without receiving information from the peripheral nervous system (PNS). On the other hand, Bard tried to determine which areas of the brain were responsible for the generation of emotions through experiments of ablation of the cerebral cortex. The proposed explanation is today known as the Cannon–Bard or thalamic theory of emotions, as it emphasizes the importance of the thalamus in emotional processing. According to Cannon and Bard’s interpretation, emotional events have two separate effects on the brain: they stimulate the ANS to elicit the physiological arousal that prepares the body to respond to a threat, and simultaneously, they cause the cerebral cortex to perceive emotions; therefore, autonomic arousal and cognitive interpretation of an emotional event are processed simultaneously but separately. According to this view, the thalamus is the main structure in which these two pathways separate, as it relays sensory information to the cerebral cortex, while simultaneously sending descending signals to the spinal cord to stimulate visceral changes that accompany an emotion.

**Figure 2:**
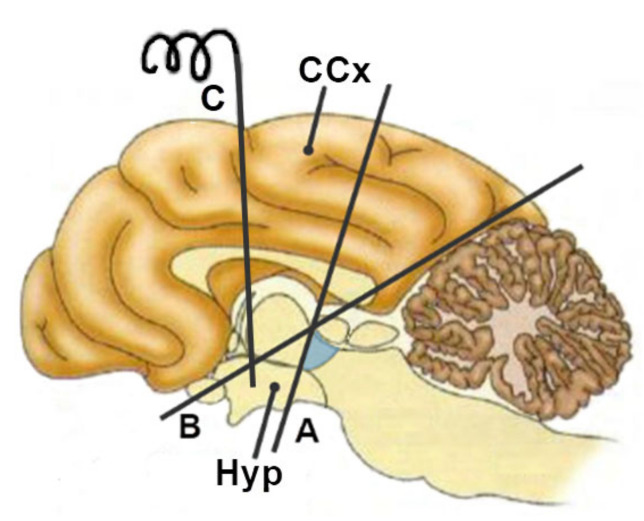
**Schematic representation of Bard’s experiments on cats.** Behavior described as “false anger/false rage” occurs if the cutting line when decorticating a cat goes from the posterior part of the cerebral cortex through the anterior part of the hypothalamus (line marked with (**B**)), but not if it goes through its posterior part (line marked with (**A**)). In both cases, a small part of the caudoventral part of the thalamus remains preserved (marked in blue). Electrical stimulation of the hypothalamus with an electrode (without cutting) leads to anger and fear (**C**). The schematic follows Bard’s textual description (Bard, 1928) [35]. CCx—cerebral cortex; Hyp—hypothalamus. See text for details.

In experiments observing the behavior of decorticated cats (“acute thalamic cats”), Cannon and Bard observed that these cats had a tendency to attack all objects in their immediate environment furiously and unreasonably, while increasing sympathetic activity yielded tail wagging, violently alternating leg twitching, back bending, claw scratching, and biting. Because such activity occurred in the absence of an externally evoked experience of anger, and could be provoked by the slightest stimulus, such as a light touch, Cannon and Britton called such behavior “a sort of sham anger/sham rage” [34]. Based on these experiments, it was hypothesized that the thalamus is responsible for expressing emotions in response to a stimulus and that the cerebral cortex inhibits the expression of emotions [35,36,37]. Further studies refuted this theory, including the importance of the thalamus in experiencing emotions. Even Bard himself in 1928 concluded that the “false rage” in cats does not occur if the cutting line by which decortication is performed goes from the posterior part of the cerebral cortex to the the anterior (line B in Figure 2), and not the posterior, hypothalamus (line A in Figure 2; in both cases a part of the thalamus is removed, Figure 2), a finding that was also confirmed by his experiments with the direct stimulation of the hypothalamus (electrode C in Figure 2) [35,36,37,38].

At the time, it was already well known that the hypothalamus, not the thalamus, is directly involved in sympathetic activation. For example, it was known that damage to the hypothalamospinal tract, a pathway whose fibers project from the hypothalamus to the sympathetic ciliospinal center in the spinal cord, leads to ipsilateral Horner’s syndrome. Moreover, in subsequent and more elaborate experiments similar to Bard’s, Hess showed that electrical stimulation of various parts of the hypothalamus in non-anesthetized cats could slow down the heart rate and make the cat calm, tame, and sleepy, speed up the pulse and cause fear and anger, cause hunger or thirst, and induce other autonomic reactions and extrapyramidal motor signs and instinctive behaviors, as well as “affective-defensive reaction”—an excited cat would attack the first available object in its environment [39]. Thus, the basic premise of the thalamic theory of emotions that physical reactions do not lead to emotions was rejected. As already mentioned, even when individuals are only asked to make a certain facial expression or speak the word for an emotion, they usually experience a fraction of the emotion associated with it. Finally, Panksepp showed in the 1980s that animals that exhibit anger-related behaviors do indeed feel anger, and therefore one cannot speak of “false anger” or “false rage” [40,41].

Schachter and Singer considered that activation of ANS acts as a signal that stimulates cognitive processes that give final meaning to an emotional state. As physiological arousal is nonspecific and relatively similar for all emotions, a subject cannot determine his/her current state and therefore activates the process of “cognitive labeling”, which recalls previous experiences related to arousing stimuli and, depending on the available information, gives different meanings to emotional states. It was shown much later that in this type of learning, the instinct plays a great role because, for example, rhesus monkeys very quickly learn to fear snakes and snake-like objects just by looking at the reactions of other monkeys, while fear conditioning is much slower for other objects (such as a flower) [42]. As Schachter and Singer were interested in situations where there is no immediate explanation for an increased level of general arousal or excitement, they designed experimental conditions in which subjects need to evaluate their own arousal in the absence of objective standards or previous experience, assuming that people would engage in social comparison as a source of information in order to minimize feelings of insecurity in situations without previous personal experience. The experiment included 185 men who were told that the experimenters intended to evaluate the effects of a small and harmless injection of a vitamin on visual abilities [43]. After determining pulse frequency, the subjects received a subcutaneous injection of half a cubic centimeter of an adrenaline solution (1:1000) or a placebo (same volume of saline) instead of “the vitamin”. Some participants were correctly informed that they actually received adrenaline to induce sympathetic activation, and those subjects felt palpitations, tremor, and redness of the face for about 15–20 min. as a direct consequence of receiving the injection; others were misinformed that their feet will tingle after the injection, they will feel itching, or they may have a mild headache, while some were not informed at all about what to expect. After receiving the injection, all participants filled out a questionnaire in a separate room where the assistant was present, and his role was not known to the participants. The assistant was instructed to pretend to be a participant given the same injection of “the vitamin” and to behave either cheerfully or angrily. As expected, the results showed that participants who were accurately informed about the effects of the injection did not experience any particular emotional experience since they knew why ANS arousal had occurred. However, some of the participants who did not know what to expect from the injection (of adrenaline) experienced feelings of either euphoria or anger that wereinduced by the assistant’s behavior. These results are partly in line with the James–Lange theory of emotions, as it states that bodily reactions are perceived as emotions, but to some extent are also compatible with that part of the Cannon–Bard theory, which assumes that the basis of different emotions lies in non-discriminatory general physiological arousal. Compared to well-informed participants, those who did not have an adequate explanation for their excitement tended to attribute it to environmental (social) factors. From the answers on the subjective experience of emotions obtained by the questionnaire and the analysis of emotional behavior of the respondents, Schacter and Singer concluded that uninformed participants who experienced physical arousal but did not know that it was a consequence of adrenaline injection attributed their physical changes depending on the behavior of the assistant. Participants who received the placebo generally did not have any particular emotional experience, regardless of the assistant’s behavior, as they did not experience activation of ANS. It was concluded that the emotional state resulted from the interaction of bodily arousal and cognitive interpretation of that arousal. This paradigm was called the two-factor theory of emotions [43]. These findings revealed that experiencing emotions is strongly influenced by cognitive processes of interpretation and evaluation, a fact now embedded in the foundations of all contemporary theories of emotion.

Arnold and Lazarus further developed existing theories of emotion. According to Arnold, emotions are the result of an unconscious evaluation of a situation, whereas feelings are a conscious reflection of that unconscious assessment, a hypothesis supported by the fact that even a subliminal stimulus can produce an emotion [44]. In contrast to all other theories, only Arnold did not hold ANS necessary for generation of an emotion. Arnold contributed to theories of emotion also by describing the three main dimensions of assessing events in the environment: whether events are potentially beneficial or potentially harmful/threatening; the presence vs. absence of an incentive/arousing stimulus; and the degree of difficulty to avoid or approach that stimulus. It is difficult to say how many and which dimensions of assessment are the most important, but later research by Smith and Ellsworth indicates eight main dimensions of cognitive appraisal in emotion: (1) attention—the degree to which someone focuses on a stimulus/situation/event and how much she/he thinks about it, (2) assessment of the probability of an outcome (to what extent an outcome is expected, or to what degree one is convinced that something will happen), (3) control/skill of managing the situation—the degree to which one can control the outcomes, or the extent to which we think we understand the current situation, predict its future development, and face its consequences, (4) comfort—the degree of positive or negative valence of a stimulus/situation/event, (5) perceived obstacles—the extent to which the goal one strives to achieve is hindered or blocked in relation to given efforts, 6) responsibility—the degree to which a person or some other factor is responsible for an event, (7) justification—the degree to which an event is fair and deserved, or unfair/undeserved, which includes compliance with personal, but also with social standards, and 8) presumed effort—the degree to which someone must spend their energy and time to respond to a stimulus/situation/ event [45,46].

According to Arnold, feelings arising from an unconscious assessment represent tendencies for action. Feelings are different, as they trigger tendencies in different situations, but they are also individually variable because the same stimulus can provoke different emotional reactions in different people. Based on some of these considerations, Lazarus developed the idea that emotions arise as a result of series of evaluations [47]. According to Lazarus, the primary assessment (appraisal) is aimed at determining the positive or negative significance of a particular event for an individual’s well-being (i.e., comfort vs. discomfort). After the primary, there is a repeated assessment (reappraisal) aimed at determining a person’s ability to cope with the consequences of an event, taking into account her/his skills, strength, experience, and other characteristics. The underlying idea of all emotion theories based on cognitive assessment is the existence of a series of continuous evaluations of stimuli within a situation, with each of these evaluations progressively leading to increasingly complex decisions. At the core of these theories is the assumption that the one’s own interpretation/assessment/opinion/memory of a situation, object, or event can contribute to the experience of different emotional states. Conforming to this understanding, assessment occurs before emotion, i.e., emotions are the result of cognitive processes. This theory is, therefore, called the cognitive–mediational theory of emotion [47], as repeated appraisal often changes or corrects first impressions and thus, also the resulting emotions.

**Figure 3:**
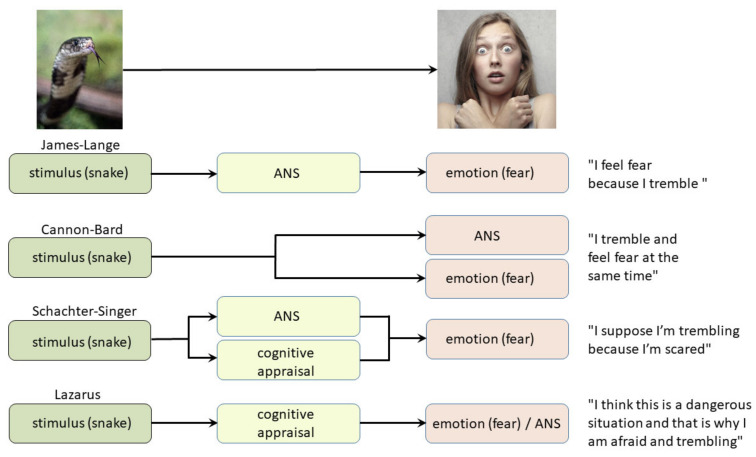
**Simplified schematic representation of classical theories of emotion.** Photographs taken from [9,48]. ANS—autonomic nervous system. See text for details.

It appears that emotions are not opposed to reason, but that they are even more fundamental, as they have the ability to guide and manage behavior, even in novel contexts and in the absence of logical thinking. A comparative overview of all four classical theories of emotions is illustrated in Figure 3.

### 2.1. Contemporary Theories of Emotions

Recently, there have been many attempts to provide a single, all-inclusive, universal theory of emotions. The most acknowledged ones are the somatic marker hypothesis, the theory of emotions as (psychological) constructions, and the higher-order theory of emotion.

#### 2.1.1. Somatic Marker Hypothesis—Interoceptive Theory of Emotions

This theory was introduced by Damasio and coworkers [20,24,49]. The term somatic implies musculoskeletal and visceral body parts, whereas somatic markers represent emotional reactions containing a strong physical or bodily component that supports the decision-making process [20]. Emotional reactions are based on a person’s experiences from previous similar situations. From earliest experiences in infancy, somatic markers continuously increase the efficiency and accuracy of decision-making, as they allow a quick overview of possible alternatives, which are then subjected to more detailed cognitive processing, leading to the final decision. As such, bodily states caused by the experience of pleasant emotions (rewards) or unpleasant ones (punishments) signal the potential occurrence of a particular outcome and guide behavior in such a way that a person chooses alternatives that bring pleasure or benefit [50]. The theory is based on observations of patients with injuries to the frontal lobe, especially with the involvement of its ventromedial part of the prefrontal cortex (vmPFC), including the well-known case of Phineas Gage. These patients show severe difficulties in making decisions and goal-oriented behaviors, either personal or social, despite having other intellectual abilities (such as attention, working memory, general intelligence, and reasoning) largely preserved. They also find it difficult to plan everyday actions, future short- and long-term goals, family and social activities [20]. Importantly, they also struggle with expressing emotions and experiencing feelings in situations where this is expected from them. Thus, besides more or less normal intellectual functioning and impaired decision-making, they have significant problems in the domain of emotional behaviors. Because they can no longer include emotions in the interpretation of complex situations, they cannot follow social norms and make decisions for their own benefit. This fact can be demonstrated, for example, by their failure on the Iowa gambling task, which serves to simulate real-life decision-making [49]. According to Damasio, such patients have a disorder of somatic markers that would otherwise help them to anticipate the consequences of their behavior and guide them to choose the most favorable decisions. Somatic markers can arise on the basis of primary or secondary emotions, where emotions have the role of inducing them, and as such, somatic markers can be understood as guides in decision-making and social behavior. In this sense, changes in somatic and visceral states would predict what individual external stimuli might cause to our body and anticipate what effects of such stimuli increase or decrease the likelihood of survival in different contexts. In uncertain situations, somatic markers will limit the number of possible choices of behavior, thus facilitating decision-making. When primary emotions occur in an environment, they automatically elicit an innate response consisting of two processes (stages): in the first stage, a specific feeling is created that has a pleasant or unpleasant valence, whereas in the second stage, somatic markers will help choose the best response among possible options (automatic emotional response). According to the theory of somatic markers, the amygdala is the key place in the CNS that triggers somatic states from primary emotions, as it matures before the cerebral cortex of the frontal lobe. These somatic markers in the amygdala form an initial repertoire of bodily responses in directing the child’s choice of reactions to a situation, while somewhat later in life the vmPFC generates secondary emotions from primary ones, as it receives information about them via the uncinate fasciculus. Magnetic resonance tractographic analysis of the microstructural maturation of the uncinate fasciculus, as judged from fractional anisotropy index, revealed that the development of this bundle of axons is longer than any other fiber system in the entire CNS, lasting at least up to 30 years of life, which correlates well with its protracted development throughout adolescence [51,52]. When it comes to secondary emotions, somatic markers are generated by OFC, especially vmPFC, which links individual situations to somatic states, meaning that these reactions are based on both the feelings and previous experiences of individual emotions.

The somatic marker theory provides a neuroanatomical framework for understanding the impact of emotions on decision-making and behavior in general [24]. Altogether, vmPFC is the key place where all somatic markers are generated from secondary emotions. The vmPFC receives projections from all sensory modalities, both directly and indirectly. This is also the only part of the frontal lobe associated with ANS that also has extensive reciprocal connections with the hippocampus and amygdala. The vmPFC mediates at least three broad domains of behavior: a reward-based decision-making process, which arises through interactions with the ventral striatum and amygdala; regulation of emotions with negative valence, which occurs through interactions with the amygdala, bed nucleus of stria terminalis (BNST), periaqueductal gray (PAG), hippocampus, and the dorsal part of the anterior cingulate cortex (ACC); and multiple aspects of social cognition, such as recognition of emotional facial expressions, ability to attribute mental states (beliefs, intentions, desires, emotions, knowledge) to oneself and others (also called the theory of mind), processing relevant self-related information through interactions with posterior cingulate cortex (PCC), precuneus, dorsomedial PFC (dmPFC) and amygdala [53]. Therefore, injury or pathological changes to the vmPFC lead to more or less serious difficulties in social behavior and decision-making, which also impairs everyday functioning. The influence of somatic markers can occur on multiple levels, both conscious and unconscious, and involves different parts of the brain: vmPFC, amygdala, somatosensory cortex, insula, basal ganglia, ACC, brainstem, as well as humoral signals and afferent pathways signaling bodily states. Primary emotions are innate and crucial at a time when the ventromedial OFC is immature. When primary emotions occur in a certain context, they automatically provoke an innate response consisting of two stages: first, a specific feeling that has either a positive (pleasant) or negative (unpleasant) valence; and second, as a separate process, somatic markers will help select the best possible response, that is, the behavior among all the possible options available at that time. These automatic responses are first controlled by the amygdala, which matures before the cerebral cortex of the frontal lobe. When it comes to secondary emotions, somatic markers are generated by the vmPFC, which categorizes and associates individual situations with somatic states, meaning that these reactions are based on both feelings and previous experiences of individual emotions. Thus, somatic markers can arise on the basis of both primary and secondary emotions, and they can be understood as inducers of certain responses that help us and guide us in decision-making and social behavior. In this sense, changes in somatic and visceral states represent anticipation of what certain external stimuli could cause to our body (harm it or be useful), so proper anticipation of the effect of such stimuli will increase the likelihood of survival in different contexts. In uncertain situations, somatic markers will limit the number of possible choices and thus facilitate and speed up making the right decisions. In conclusion, the somatic marker theory proposes that the amygdala mediates somatic markers as a response to generated primary emotions, whereas vmPFC is a key hub where features of a given external stimulus are converted into the visceral states associated with the biological importance of that stimulus [54].

The somatic marker hypothesis shares certain features with the James–Lange theory of emotions, such that feelings and conscious experience generally arise from the representation of bodily states embedded and distributed across multiple areas and levels of the nervous system, including cortical and subcortical structures [55]. The somatic marker hypothesis further assumes that the representation of the body is necessary not only for emotions [56], but also for a broader core self-image, crucial for feelings to arise [4], which is in agreement with the notion that conscious experience cannot occur without feelings and interoception [57,58]. While presenting an elegant theory of how emotion influences decision-making, the somatic marker hypothesis requires additional empirical support to remain tenable in regard to psychopathic traits, moral decision-making, and other issues [59].

#### 2.1.2. Theory of Constructed Emotion

The psychologically constructed emotion theory was proposed by Feldman Barrett [60,61,62]. The initial assumption is that the brain creates internal models based on experience, and uses them to predict future events, chooses the best actions to deal with upcoming situations and anticipate their consequences. Information that the brain has not predicted (prediction error) is coded and consolidated whenever it results in physiological changes. Once the prediction error is minimized, prediction becomes perception or experience. Thus, prediction explains the causes of sensory events and directs further action.

Accordingly, the brain constantly constructs concepts and creates categories with the goal of identifying input sensory information, drawing conclusions about causes, and implementing action plans, whether or not a person is consciously focused on them. When an internal model creates an emotional concept, its eventual categorization results in an emotional episode (“instance of emotion”). Feldman Barrett assumes that certain categories of emotions do not have a specific substrate that can be unambiguously localized in precisely defined areas of the brain, as judged from strongly diverged activations among studies that investigated the localization of anger, happiness, sadness, and disgust [62,63]. Even someone with isolated damage to the amygdala (such as patient S.M., see below) can correctly recognize fearful faces when his/her attention is directed toward the eyes of the stimulus face, which is due to the fact that the eyes are the most important feature for identifying this emotion [64]. In favor of this view, it should be added that inhalation of 35% CO_2_ evoked fear and panic attacks in three patients with bilateral amygdala damage, indicating that the amygdala is not required for fear and panic, making an important distinction between fear triggered by external threats from the environment versus fear triggered internally by CO_2_ [65].

Emotion categories are as real as any other construct that requires awareness to exist. According to the theory of constructed emotion, emotions such as fear, anger, or sadness are socially and experientially constructed categories and therefore, vary with culture and time [4]. In neuroscientific jargon, construct refers to a group of distributed activity patterns of specific neuronal populations. An individual emotion is constructed in the same way as all the other perceptions, through information flow within neural circuits. Consequently, the brain neither specializes in processing emotions nor are emotions innate. Instead, it is the innate ability of the brain to create assumptions or predictions to construct an emotional episode depending on a given situation, as is so for many other general processes related to a particular domain (e.g., memory, perception, or attention) [4]. In other words, the relationship between the brain and emotions should be observed through a prism of the understanding that a given brain structure or area can have multiple functions, depending on the currently active functional network and co-activation patterns in all active areas at a given time [66].

The internal model that the brain creates to maintain allostasis is at the heart of the constructed emotion theory. Allostasis, unlike homeostasis, refers to the effective allocation of resources for changing the physiological and behavioral systems within an organism to achieve homeostasis, so that the organism can grow, survive, and reproduce [67]. Allostasis is not a body state, but a process through which the brain regulates bodily functions according to cost/benefit criterion, requiring the ability to anticipate future bodily needs and meet them before they arise [67]. The brain monitors many variables and integrates their values with previous knowledge and experience to anticipate needs and set priorities. As such, the brain is not a passive organ responding only to input signals and acting on the basis of the negative feedback principle (as is the case with most homeostatic mechanisms), yet it actively constructs perceptions based on internal models, predicting future input signals and calculating prediction error (i.e., differences between predictions and input signals).

According to Sterling’s allostasis model, the design of efficient predictive regulation depends on the brain’s ability for sensing the current state, integrating this information with prior knowledge to optimize regulatory decisions, and on relaying current sensory information to higher-order brain levels so that today’s learning becomes tomorrow’s “prior knowledge” [67]. In his “carrot and stick” model of allostatic anticipatory regulation, the “carrot” component is the midbrain reward system, whereas the “stick” component is the amygdala, as it integrates a large number of lower level physiological signals from the entire body, such as steroid hormones and peptides that regulate blood pressure, hypothalamic and brain stem signals containing visceral information (e.g., from the nucleus of the solitary tract), and signals from serotonergic neurons of the raphe nuclei of the PAG that modulate arousal levels and mood [67]. The amygdala is heavily and reciprocally connected with the hippocampus and vmPFC and these pathways provide a constant flow of information on needs and past dangers to design a plan of action. Figuratively speaking, the amygdala reports its “concerns” to the PFC, which decides what to do and performs planning for the future [67]. As posited, especially by Friston, the brain is, therefore, an organ intended for predictive regulation, the active prediction and interpretation of input sensory information [68]. The theory of constructed emotion is based on the concept of predictive coding, which assumes that the brain is an interface that creates internal models at different functional levels and that any function of the brain (perception, cognition, emotion) arises from, comparing the current model and input sensory signals [69]. In regard to interoceptive feelings, expectations and predictions of one’s own bodily states make a significant part of conscious emotional experience [4,69]. However, according to the constructed emotion theory, the key difference from Damasio’s assumptions is that the brain creates emotions from predictions that subsequently trigger physical events in the body (and not the opposite, as is assumed by the somatic marker theory).

Feldman Barrett explains that the primary, innate emotions in the first six months of life arise from physiological processes and interoception. According to the theory of constructed emotion, these states, however, should not be marked as emotions, as they are simply information about the state of bodily functions that contain insufficient detail for a child to act upon in the first six months of life. The child will be able to act purposefully (of its own volition) only with the maturation and activation of the corticospinal tract, a process which begins at about 6 months of age. More precisely, according to this theory, emotions are just brain predictions that connect bodily states to events in the environment so that the person knows how to (re)act. Only sometimes, as a by-product of these predictions, emotions arise.

#### 2.1.3. Higher-Order Theory of Consciousness and Fear Conditioning

The basic idea underlying higher-order theory of consciousness developed by LeDoux is the existence of a general cortical system (higher order) responsible for generating conscious experience from information received from first-order networks [4,70,71,72]. For example, in the case of visual information, a person becomes aware that she/he is seeing something; if it is information sent by subcortical, lower-order structures, such as the amygdala, the person becomes aware of an emotion, generally called a feeling. LeDoux hypothesizes that objectively measurable behaviors and physiological responses are driven by emotional stimuli controlled by subcortical first-order circuits, including the amygdala (unconscious or implicit level), while subjective emotional experience results from cortical higher-order circuit activities, especially involving the vmPFC, rostromedial (rmPFC) and dmPFC and OFC, but also the dorsolateral PFC (dlPFC) involved in working memory and related higher cognitive functions [73,74].

LeDoux defines fear as a feeling that enters a person’s consciousness and also bases his higher-order theory of consciousness on this subjective cortical experience [75] in the presence of danger, whether it is real or potential [72]. The human brain is capable of anticipating threatening events, even those that are unlikely to ever happen. The individual recognizes fear in oneself as an internal experience, and in others as external associated manifestations, such as freezing, escaping, trembling, frightened facial expressions, etc. In evolutionary terms, fear is associated with the activation of neural circuits responsible for survival [10].

**Figure 4:**
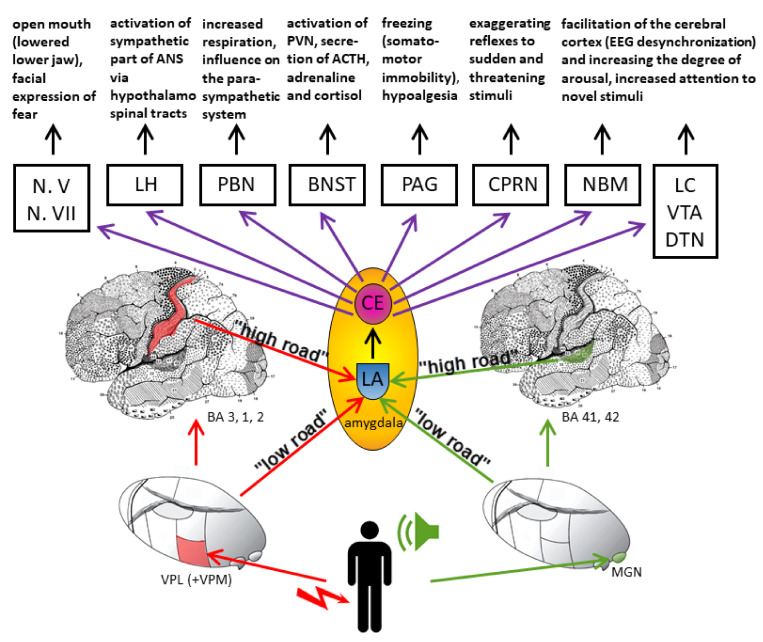
**Simplified schematic representation of neural circuits underlying fear conditioning.** Pathways that process a conditioned stimulus (CS, auditory pathway, green) and an unconditioned stimulus (US, spinothalamic anterolateral pain pathway, red) via the ventroposterolateral (VPL) and ventroposteromedial (VPM) nuclei and the medial geniculate body (MGN) of the thalamus monosynaptically, and via the cerebral cortex of Brodmann’s areas 3, 1, and 2 (primary somatosensory cortex); 41 and 42 (primary auditory cortex) polysynaptically converge on the lateral nucleus of the amygdala (LA, the LA receives the majority of afferent fibers). CS-US convergence in LA initiates long-term potentiation (LTP), leading to the creation of a learned association between the two stimuli. LA activity is then transferred to the central nucleus (CE, the central nucleus of the amygdala), which sends most of the efferent projections to a number of different cortical and subcortical areas through which the amygdala directly regulates autonomic responses and context-dependent behavior: ANS, reflexes, and hormone secretion. Sympathetic activation includes mydriasis, tachycardia, hypertension, peripheral vasoconstriction, cessation of peristalsis, sphincter contraction, and other effects. All these effects help organisms to cope with threat. Synaptic plasticity also changes in neurons in other nuclei of the amygdala (intentionally omitted here). ACTH—adrenocorticotropic hormone; BA—Brodmann’s area; BNST—bed nucleus of stria terminalis; CPRN—caudal pontine reticular nucleus; DTN—dorsal tegmental nucleus; EEG—electroencephalogram; LC—locus coeruleus; LH—lateral hypothalamus; MGN—medial geniculate nucleus; NBM—nucleus basalis Meynerti; N. V—trigeminal nerve; N. VII—facial nerve; PAG—periaqueductal gray; PBN—parabrachial nuclei; PVN—paraventricular nucleus; VPL and VPM—ventroposterolateral and ventroposteromedial thalamic nuclei; VTA—ventral tegmental area. The schematic is made according to LeDoux [73,74].

Fear conditioning is an example of associative learning, a process by which the brain creates memories about the relationship between two events (Figure 4). In a situation of fear-conditioning, an experimental animal receives a neutral conditioned stimulus, usually a sound, followed by an aversive unconditioned stimulus, such as an electric shock to the paw. After one or more pairings, the conditioned stimulus elicits a conditioned emotional response that occurs naturally in the presence of a dangerous, threatening stimulus, such as a predator. The conditioned emotional response includes changes in behavior and ANS as well as in hormonal activity induced by the conditioned stimulus. Fear conditioning is also used to examine the brain mechanisms of implicit learning and memory in animals and humans.

Studies in humans have confirmed the key role of the amygdala in fear conditioning as well as in various forms of psychopathological behavior [13]. Thus, damage of the amygdala in humans disables fear conditioning, while reduced volume of the right amygdala, along with reduced volume of BNST and other associated structures, have been documented in some sexual offenders [76]. However, the amygdala does not function independently of other structures, but is part of larger neural circuits involving sensory systems, the motor system, the hippocampus (that provides contextual information) and the PFC (responsible for regulation of amygdala reactivity, so that hypofunction of the PFC will lead to amygdala hyperreactivity). The amygdala contributes to these fear circuits in two ways: directly, by detecting the threat on an unconscious level and regulating behavioral and physiological responses, and indirectly, through cognitive systems, in the emergence of a conscious feeling of fear. Moreover, there are two main afferent pathways that lead to the amygdala: a faster “low-road pathway” that reaches the amygdala directly from the sensory nuclei of the thalamus without prior cortical processing (without reaching the level of consciousness) and activates the amygdala in a 12 ms time-frame, and a slower “high-road pathway” that activates the amygdala through the thalamus and cerebral cortex [73,74,77]. The low-road/high-road dichotomy is supported by studies of nonconscious processes in healthy subjects using magnetoencephalography (where early recorded, low-road amygdala activations upon emotional stimuli occurred after 40–140 ms, whereas later, high-road amygdala responses were recorded after 280–410 ms, subsequent to frontoparietal cortex activity, this time also being modulated by the attentional load) [78], blindsight patients [79], and in patients with electrodes implanted in the amygdala during preparation for treatment of epileptic seizures [80].

LeDoux (2002) illustrated the independence of emotional processing from the conscious control of emotional behavior by stating that the feeling of fear appears only after an individual has unconsciously reacted to the perceived threat and changes in ANS have occurred. He used the term “fear system” to describe the whole process, including the role of the amygdala in controlling the fear response, but also in providing elements that indirectly contribute to the creation of a conscious feeling of fear [73]. More recently, LeDoux stated that he was wrong when using the term “fear system” to describe the role of the amygdala in both detecting and responding to danger because it is now commonly accepted that the term “fear” is used only to describe the conscious feeling that occurs when a person is frightened. Therefore, LeDoux proposed a new reconceptualization of the phenomena involved in the emergence and study of emotions [71,72]. Despite the proposed changes in the conceptualization and understanding of the concept of emotion, the results of the studies that LeDoux and his coworkers conducted are important links for understanding defensive behavior in animals and humans and provide a basis for understanding the occurrence of pathological fears associated with increased reactivity of the amygdala and the development of anxiety disorders [81]. The proposed reconceptualization revolves around the idea that the amygdala is of paramount importance when it triggers physiological responses to threats nonconsciously [82,83] but of only relative (minor) importance when it comes to subjective feelings. Typically, direct electrical stimulation of the amygdala reliably elicits physiological responses, but subjects do not report feelings, even when asked for a verbal report [84,85]. Moreover, patients with lesions of the amygdala can consciously report emotional experiences, including fear [65,86].

According to the higher-order theory of emotion, a subjective experience of emotions should be generally different in subjects with damage to the first-order circuits associated with emotions (patients with amygdala damage) and patients with damage to higher-order circuits associated with emotions, such as patients with alexithymia, for example, but this remains to be determined.

## 3. The Structure of the Amygdala

**Figure 5:**
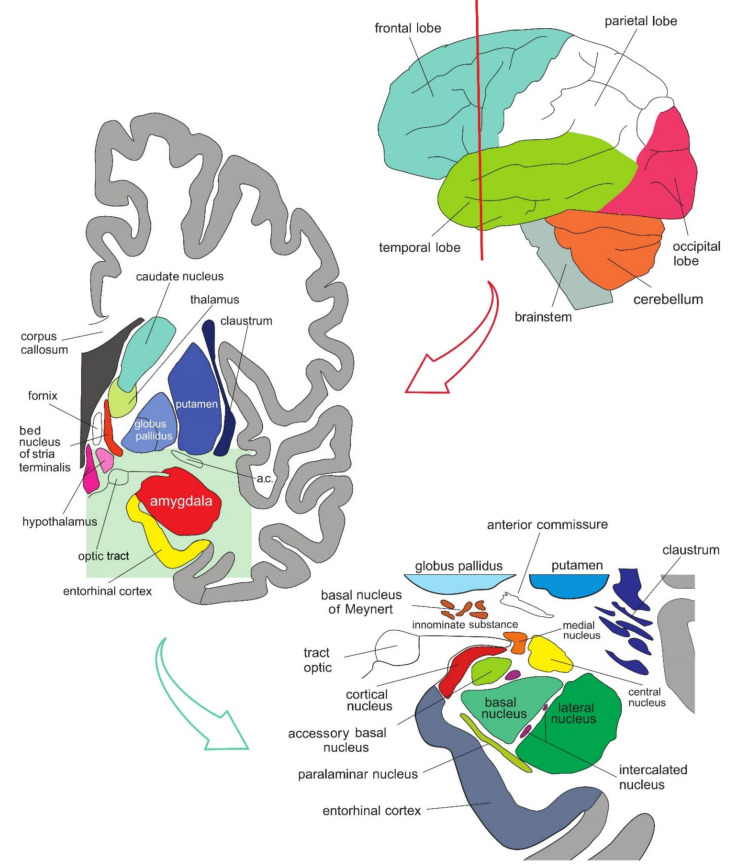
**Simplified representation of the structure and location of the amygdala.** The upper part of the schematic shows the human brain when viewed from the lateral side, where the brainstem, cerebellum, and four lobes of the cerebrum can be seen. The middle part of the schematic shows the structures present on the coronal plane through the temporal lobe of the cerebrum on which the position of the amygdala can be observed. The lower part of the schematic shows an enlarged amygdala with its individual nuclei. a.c.—anterior commissure. See text for details.

The amygdala is formed by several nuclei and cortical fields located bilaterally in the anteromedial part of temporal lobes of the cerebrum (Figure 5). There are several concepts about what the term amygdala should encompass as well as whether it is a single structure or a set of extensions from different parts of the brain [87].

**Figure 6:**
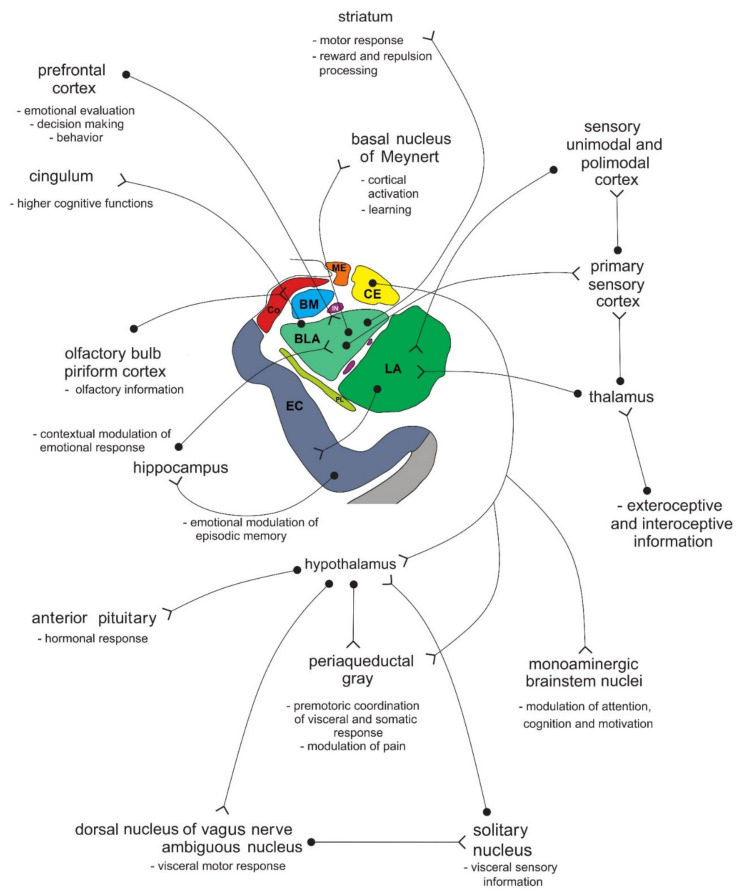
**Simplified schematic representation of the connections of individual amygdala nuclei with numerous cortical and subcortical structures, and their role in processing functionally different types of information.** Amygdala nuclei are marked in colors as shown in Figure 5. BLA—basolateral (basal) nucleus; BM—basomedial (accessory basal) nucleus; CE—central nucleus; Co—cortical nucleus; EC—entorhinal cortex; IN—intercalated neurons; ME—medial nucleus; LA—lateral nucleus; PL—paralaminar nucleus. See text for details.

In primates, the amygdala is usually divided into 13 nuclei and cortical fields [88,89,90,91]. Most agree that the amygdala can be divided into several groups of nuclei, as some nuclei show certain anatomical and functional similarities. The deep or basolateral group contains the lateral, basal, accessory basal and the paralaminar nucleus. The superficial or corticomedial group includes the cortical nucleus in contact with the relatively thin periamygdaloid paleocortex, the central and medial nuclei as two functionally similar nuclei, and the nucleus of the lateral olfactory tract, which some authors do not include as a part of the amygdala. The BNST might be added to this group although most do not consider it a part of the amygdala. It should be noted that the central nucleus (CE) has a more specific functional role and connections, so it can be observed separately. Additional nuclei include the anterior amygdaloid area, the amygdalohippocampal area, and groups of inserted neuronal clusters (Figure 6).

### 3.1. The Lateral Nucleus (LA)

The lateral nucleus (LA) extends across the entire length of the amygdala. It is the largest nucleus of the human amygdala [92] with a high density of nerve cells [93]. The LA is extremely well connected intrinsically (its individual parts are interconnected) as well as with other nuclei of the amygdala, mostly with the basal nucleus [94]. It receives poor reciprocal projections from other nuclei, mostly from the basal, accessory basal or central nuclei [95]. The lateral nucleus is also the main afferent structure of the amygdala, and as such, receives topographic projections from various neocortical fields. These signals are then transmitted both to other amygdala nuclei and other parts of the lateral nucleus [77,96]. Glutamatergic projections are sent to central and medial as well as basomedial and basolateral nuclei [97]. Consequently, information flow through the amygdala proceeds from lateral to medial parts [98]. Weak projections from the LA and CE also end in the amygdalohippocampal area on small to medium-sized neurons [95].

### 3.2. The Basolateral Nucleus (BLA)

The basolateral nucleus (BLA, more commonly called just the basal nucleus), contains the largest neurons of the amygdala and is also called the “cortex within the amygdala” [99], as those pyramidal neurons share many morphological characteristics and immunohistochemical profiles with cortical pyramidal neurons [100]. The majority of afferent fibers in BLA come from the LA [101,102]. The BLA sends the majority of efferent projections toward the OFC, mPFC, and ventral striatum, with the nucleus accumbens (NAc) as the largest targeted group of neurons [103]. The BLA receives the strongest projections from the LA, and further sends processed information to the CE. It is important to note that the BLA sends projections to a number of cortical areas that project to the LA [104], forming sensory information flow loops between the amygdala and cerebral cortex [105]. The intrinsic activity of different populations of GABAergic interneurons determines the output activity of efferent pathways from the amygdala [106]. In the BLA, fear and reward are encoded by phasic activation of distinct populations of neurons, while anxiety results in persistent activity changes [107]. Likewise, different groups of neurons are involved in consolidating the memory of objects, situations, and events that elicited the fear response (the feeling of fear), thus mediating fear conditioning [108]. The direct manipulation of the amygdala neural circuits in rodents by usage of optogenetic and pharmacogenetic activation or inhibition, in conjunction with behavioral and electrophysiological analysis, revealed causal relations between different cell types, especially in the BLA, and their projections, which are sufficient to alter behavior in various domains (freezing, anxiety, feeding, social behavior) [103]. The activity and synaptic connections within populations of GABAergic neurons change depending on life experience, which helps in understanding and explaining how different, earlier events shape current behavior.

### 3.3. The Basomedial Nucleus (BM)

The basomedial nucleus (BM) is also known as the accessory basal nucleus. Topographically, it represents a bridge between the BLA and CE [109]. It mostly projects into the CE, especially its medial part [110]. Neurons in the BM secrete a variety of peptides, such as corticotropin-releasing hormone/factor (CRH/CRF), enkephalins, and neurotensin, and express dopaminergic and serotonin receptors [111]. Interestingly, these neurons express estrogen receptors as well. Therefore, this area is thought to play an essential role in shaping motivational behavior under the influence of sex hormones.

### 3.4. The Amygdalohippocampal Area

The amygdalohippocampal area represents the most caudal part of the amygdala. Most internal connections come from the LA, BLA, BM, medial, and CE nuclei [95,110,112]. Projections from this area seem to terminate in the BLA, medial nuclei, and the periamygdaloid cerebral cortex [113]. The anterior amygdaloid area is poorly developed in primates and also poorly connected to the other nuclei.

### 3.5. The Paralaminar Nucleus (PL)

The paralaminar nucleus (PL) is a narrow band of densely packed neurons along the ventral and rostral boundaries of the amygdala, mostly along the BLA (basal nucleus). It is characterized by a high density of neurons resembling glia and non-pyramidal neurons [100,114]. A relatively high concentration of CRH receptors and benzodiazepine receptors has been demonstrated in this nucleus, as well as abundant innervation with serotonin fibers [115,116]. Paralaminar nucleus receives afferent fibers mainly from the LA [112], whereas it projects into the BLA (basal) nucleus [94,113].

### 3.6. The Intercalated Neurons (IN)

The intercalated neurons (IN) correspond to a small group of nerve cells located in internuclear fibrillar areas, mostly in the rostral parts between the BLA (basal) and the BM nuclei. These are mainly interneurons, with GABA as their principal neurotransmitter, and they also abundantly express dopaminergic D_1_ and opioid μ receptors [117,118]. Despite the relatively small number of cells and their dispersion, their role is extremely important [119,120]. The neural circuits in which they participate receive direct projections from the OFC, LA, and BLA (basal) nuclei, and project into the CE [121], where they exert an inhibitory effect. Their critical role is reflected in their activity, which alleviates the physiological response to fear, acting through inhibition of the CE [122].

### 3.7. The Central Nucleus (CE)

The central nucleus (CE) is the main source of efferent fibers of the amygdala; it shows many similarities with the striatum in the basal ganglia [123]. Over 90% of neurons in the CE are GABAergic [124,125,126,127]. The medial part of the CE receives glutamatergic projections from the BM, whereas the lateral part receives GABAergic input from the medial nucleus [128]. GABAergic neurons in the lateral part of the CE express a variety of neuropeptides that act as neuromodulators [129]. These peptides are thought to be produced only in conditions of pain [129,130] or stress [124,131], but not in normal circumstances. They can be divided into those that amplify (i.e., CRH/CRF, dynorphin, orexin, vasopressin) and those that reduce (i.e., oxytocin (OXT), neuropeptide Y, nociceptin, and other endogenous opioids) anxiety and pain [124]. In addition to modulating the affective features of pain, pain can be further reduced by enhancing the descending activity of the endogenous analgesic system (mostly raphespinal projections from the caudal group of serotonin raphe nuclei B1 (nucleus raphe pallidus), B2 (nucleus raphe obscurus) and B3 (nucleus raphe magnus)) to inhibitory interneurons of the posterior horn neurons of the spinal cord that control the entrance of nociceptive signals (Melzack and Wall, gate control theory) [132]. Additionally, oxytocin is a great pro-social hormone, as it increases cooperation and connection with other people and domesticated animals, especially dogs and horses [133,134]. The CE, after the hypothalamus, contains the highest density of CRH/CRF in its GABAergic neurons [135]. In short, considering the overall abundance of receptors for modulating neurotransmitters, hormones and various peptides, it can be concluded that many neurotransmitters and hormonal systems affect the activity of the amygdala and its role in emotional processing [136]. Activation of the lateral part of the CE, which projects to PAG in mice, produces characteristic freezing behavior in situations of real or perceived danger and also mediates a significant part of other bodily reactions in the fear response [137]. GABAergic projections from the lateral part of the CE also exert strong influence over the hypothalamus and brain stem [110,129]. Even though the corticomedial area is phylogenetically older [138], in sharp contrast to rodents, the BLA in primates is significantly larger than the corticomedial area. This is presumably due to dense reciprocal connections between the BLA and the cerebral cortex [103]. The LA influences the CE directly, through excitation by glutamatergic projections, and indirectly, through GABAergic neurons [129]. Interestingly, the LA is not directly connected to the corticomedial area, but only to the central part of the CE [95,103]. Thus, input signals are always pre-processed before exiting the amygdala. Therefore, the CE can be thought of as having a unique role in converting sensory information into a physiological response and behavior change [124].

### 3.8. The Medial Nucleus (ME)

The medial nucleus (ME) of the amygdala can be considered to be in conjunction with the cortical nucleus with which it shares a laminar structure and also with the CE, with which it partially shares the functional role. It also mostly contains GABAergic neurons [127]. As is the case with other nuclei of the amygdala, activation of the ME is also associated with psychological stress, which, in turn, leads to activation of the hypothalamic–pituitary axis and secretion of ACTH (Figure 4).

### 3.9. The Cortical Nucleus (Co)

The cortical nucleus (Co), as the entire superficial group of amygdala nuclei, is directly connected to the olfactory system and participates in the processing of olfactory stimuli [139,140]. The nucleus of the lateral olfactory stria is relatively smaller in primates than in rodents and other mammals, and has three layers, just like all nuclei of the superficial group [139,141]. It seems to be poorly connected to other nuclei, and plays the role of olfactory processing [142,143].

### 3.10. The Periamygdaloid (Prepiriform) Cortex

The periamygdaloid (prepiriform) cortex is sometimes called the corticoamygdaloid or amygdalopiriform transition area. Due to the heterogeneity of this paleocortical region, there have been many attempts for its classification [90,144,145]. It receives olfactory projections directly from the olfactory bulb as well as indirect projections from the piriform cortex [139,146]. It projects into the LA and receives weak intrinsic projections from the BM, medial, and CE nuclei [90,110,147]. 

**Figure 7:**
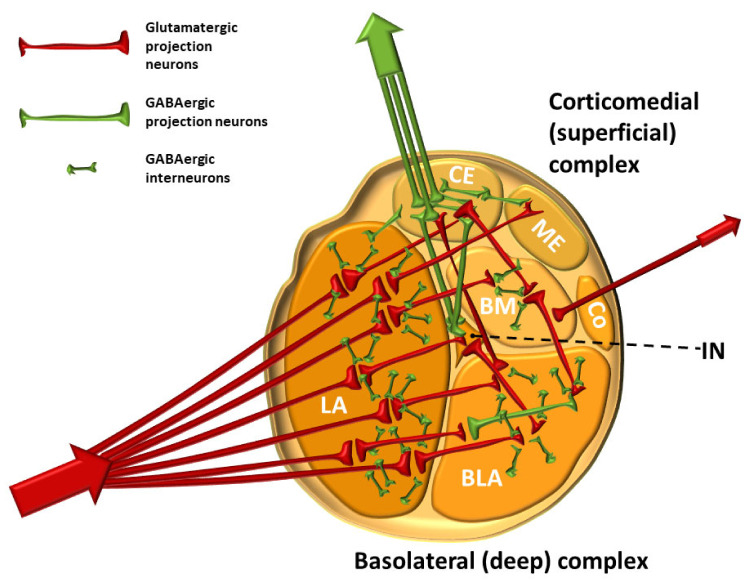
**Simplified neuroanatomical representation of information flow within the amygdala.** BLA—basolateral nucleus of the amygdala; CE—central nucleus of the amygdala; Co—cortical nucleus of amygdala; IN—intercalate neurons; LA—lateral nucleus of amygdala; ME—medial nucleus of amygdala; BM—basomedial (accessory basal) nucleus of the amygdala. The schematics is made according to Wieronska et al., (2010) [148], Orsini and Maren (2012) [111], Benarroch (2015) [149], Gilpin et al., (2015) [116], Janak and Tye (2015) [147], and Sangha et al., (2020) [80].

A simplified flow of information through the amygdala from the neuroanatomical perspective is schematically shown in Figure 7.

**Figure 8:**
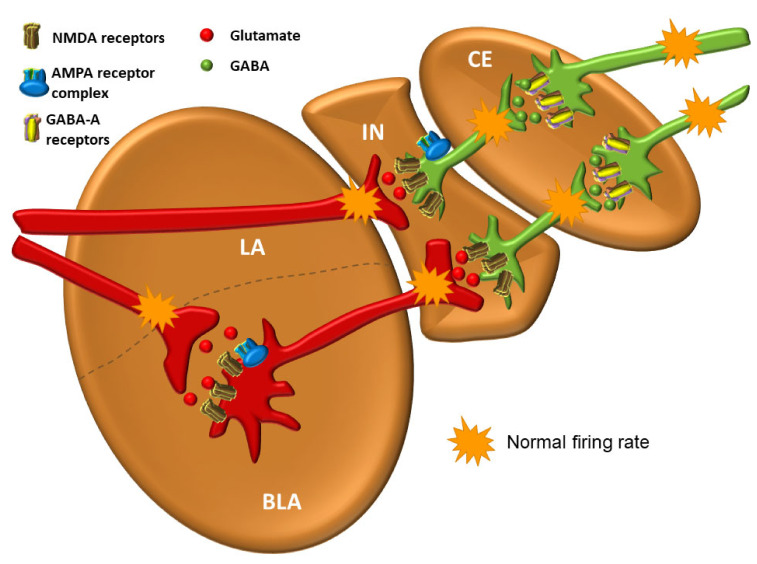
**Balanced ratio of excitation and inhibition in amygdala in a healthy individual in a non-threatening situation.** AMPA—α-amino-3-hydroxy-5-methyl-4-isoxazole propionic acid; BLA—basolateral nucleus of the amygdala; CE—central nucleus of the amygdala; GABA—γ-aminobutyric acid; IN—intercalated neurons; LA—lateral nucleus of amygdala; NMDA—*N*-methyl-D-aspartate.

The balance between excitation and inhibition determines the overall degree of amygdala excitability. The BLA complex consists of 80% pyramidal, glutamate neurons, while 20% are GABAergic [111]. Although GABAergic neurons are fewer in number, they normally exert effective control over excitatory neurons and modulate the response to anxiogenic stimuli (see below) [129,150]. The balance between excitation and inhibition thought to be present in a healthy person under non-threatening circumstances is shown in Figure 8.

**Figure 9:**
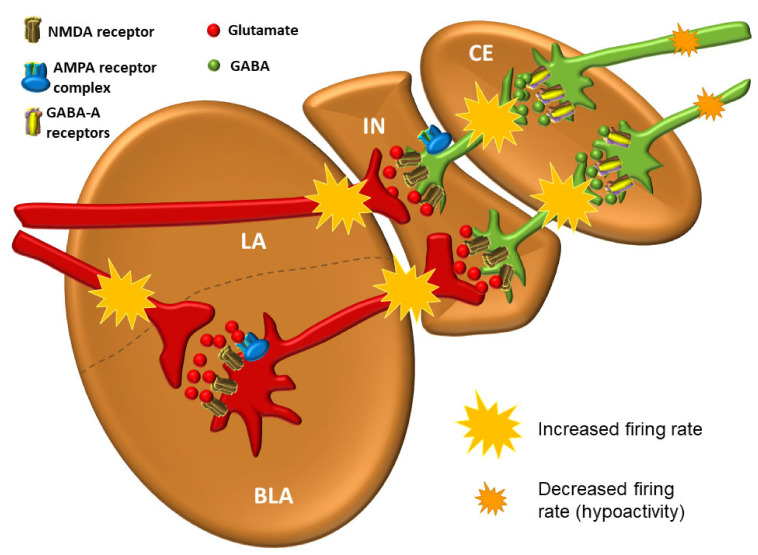
**Schematic representation of the predominance of excitation over inhibition in circumstances of imminent danger, but also in anxiety and other functional disorders of the amygdala.** The central nucleus of the amygdala contains different populations of GABAergic neurons. This area mediates inhibitory control over the lateral region of the amygdala [111]. AMPA—α-amino-3-hydroxy-5-methyl-4-isoxazole propionic acid; BLA—basolateral nucleus of the amygdala; CE—central nucleus of the amygdala; GABA—γ-aminobutyric acid; IN—intercalated neurons; LA—lateral nucleus of amygdala; NMDA—*N*-methyl-D-aspartate.

The neural network of the amygdala is very dense with high synaptic density per neuron. Hypoactivity of GABAergic neurons and/or increased activation of glutamate neurons lead to amygdala hyperexcitability that manifests as anxiety [98]. One of the key features of anxiety disorders is the inability to suppress fear appropriately in situations that do not pose a real danger [102]. All other neurotransmitter and neuromodulator systems in amygdala modulate the activity of GABAergic and glutamate neurons. Activation of GABAergic neurons in the output part of the CE results in inhibition of the physiological response and vice versa [151]. However, excitation of GABAergic IN neurons by glutamate projections from the LA results in inhibition of GABAergic neurons in the CE, ultimately leading to an enhancement of the physiological fight-or-flight response. Such an effect of one group of GABAergic neurons upon the other is called disinhibition. It is believed that the stressors that lead to excitation of the amygdala, whether it is “normal” excitation in healthy individuals or excessive excitation in various disorders, cause a decrease in the activity of projection GABAergic neurons coming out of the CE of the amygdala and consequently lead to the disinhibition of the hypothalamic–pituitary axis, as well as the disinhibition of a series of nuclei in the brainstem that are under strong influence of these pathways [151] (Figure 9).

## 4. Connections of the Amygdala

The amygdala is reciprocally connected to many cortical and subcortical areas via different fiber bundles, four of which seem to be most important: the lateral olfactory bundle, the stria terminalis, the posterior part of the anterior commissure, and the ventral amygdalofugal pathway, which also includes the ansa peduncularis [152]. The importance of the projections of the lateral olfactory bundle into the amygdala lies in the fact that they mediate the unconscious, but, unlike other sensory systems, there is a direct influence of olfactory information on the generation of emotions. Projections from various brain regions enter the amygdala through the lateral nucleus, which serves as the main entering point to the amygdala [129,153]. The LA receives excitatory input from glutamate neurons that stimulate postsynaptic glutamate *N*-methyl-D-aspartate (NMDA) and α-amino-3-hydroxy-5-methyl-4-isoxazolepropionic acid (AMPA) receptors on both glutamate and GABAergic neurons. In a simplified view, the activity of amygdala at base levels is the result of balanced inhibitory and stimulatory inputs [129]. Furthermore, the LA is thought to have a key role in the consolidation and reconsolidation of fear memories, which can be prevented with NMDA receptor antagonists [109,154] or the protein synthesis inhibitor anisomycin, respectively [155].

In all primates, the largest afferent fibers in the amygdala come from the associative cortical fields of the ventral visual pathway that provides processed information about objects and faces. This information arrives in the lateral nucleus where it is evaluated together with information from other sensory modalities to determine whether it is a known stimulus or a potential threat based on previous experiences. Visual and auditory information come topographically separated into the lateral nucleus, which enables a faster response to danger, but also indicates that stimuli from all the senses are not required to cause a fear response [156]. Likewise, these sensory inputs may be further summed up in the LA neurons (temporal and spatial synaptic integration), which may lead to a delayed response in cases when the stimulus is too weak to cause immediate activation of the amygdala and a change in behavior [157]. 

**Figure 10:**
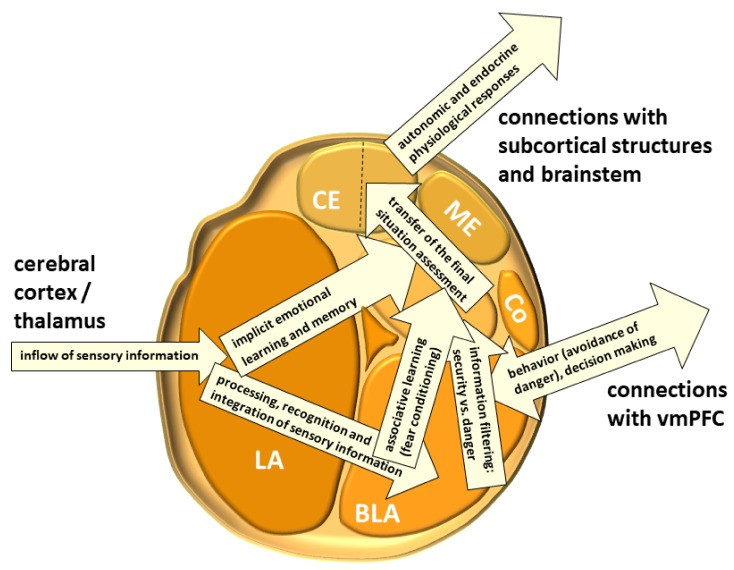
**Simplified representation of the information flow within the amygdala.** BLA—basolateral nucleus of the amygdala; CE—central nucleus of the amygdala; Co—cortical nucleus of amygdala; LA—lateral nucleus of amygdala; ME—medial nucleus of amygdala; vmPFC—the ventromedial prefrontal cortex; BM—basomedial (accessory basal) nucleus of the amygdala. The schematics are made according to Sah et al., (2017) [108], Asami et al., (2018) [162], and Neugebauer (2020) [135].

After passing the LA, sensory signals are processed in virtually all parts of the amygdala (BLA, CE, IN, etc.) and the generated information is further integrated with various other afferent signals. Conditioned impulses then leave the CE and BM. The CE is considered to provide the main efferent projections of the amygdala, including those to the BNST and PBN [158,159]. However, efferent projections, especially to the neocortex, hippocampus, and ventral striatum, emerge from the BLA and BM as well. In a simplified view, the CE “converts” emotionally important sensory stimuli into a physiological response (changes in heart rate, changes in blood pressure, sweating, tremor, and somatic sensations) and modulates behavior. The final response is the result of processing of much complex and context-dependent information, which is provided by the hippocampo–entorhinal input (see below) [99]. Using the method of retrograde transport of horseradish peroxidase in cats, Russchen and Lohman were among the first to show that the entorhinal cortex projects into the nuclei of the amygdala. They found that neurons of the deep layers of the entorhinal cortex send axons to the CE and BLA [160]. This projection is topographically organized: the medial parts of the entorhinal cortex are projected onto the medial parts of the CE and BLA, while the lateral parts are projected into the lateral parts of these nuclei. Russchen and Lohman showed that the layer II neurons of the entorhinal cortex project to the corticomedial nuclei of the amygdala [160]. In rats, a population of pyramidal neurons of layer III and layer IV of the entorhinal cortex send their axons to the periamygdaloid cortex [161]. Altogether, it is not surprising that the amygdala is compared to an interface between the frontal cortex and the hippocampus/entorhinal cortex. A simplified flow of information through the amygdala is schematically shown in Figure 10.

In addition, other brain areas and circuits that regulate the activity of the amygdala should also be appreciated. Unprocessed, direct information from the thalamus requiring immediate response represents a direct pathway crucial for prompt reaction to danger before the information has reached consciousness (the low-road pathway). Corticostriothalamic circuits regulate the flow of signals that reach the amygdala after being consciously and contextually processed and perceived (the high-road pathway) [105]. Hippocampo–entorhinal circuits also provide information about the context in which the fear occurred, which includes previously memorized information about previous encounters with the stimulus and similar contexts experienced, structurally (mainly) connected to the BLA and vmPFC. Activation of those pathways can also contribute to anxious behavior [109,163]. Finally, the main connections between the amygdala and hippocampo–entorhinal circuits run through the fornix and stria terminalis [138,164,165].

Conscious processing requires time, slowing down the flow of information through the high-road pathway. The vmPFC, ACC, and the dlPFC are thought to monitor the cessation of fear-inducing stimuli, thus regulating amygdala activity. More specifically, the vmPFC integrates emotional and cognitive information, and has an important role in decision-making and behavioral intertemporal choices, whereas dlPFC, as the end point for the dorsal visual pathway, is critical to carry out working memory, especially in remembering previous sensory events as well as attention maintenance and planning responses to emotional stimuli [166]. The vmPFC has an inhibitory effect on the amygdala, and reduces the reaction to the stressor/stressful event, as well as having an excessive emotional reactivity. Therefore, greater vmPFC activity means greater conscious effort and suppressed activity of the amygdala, along with a tendency to evaluate external experiences positively [167]. Optimal emotion regulation is thought to arise from the balance between the PFC and amygdala activity [168]. Stress negatively affects this activity of the PFC, which explains why a strategy of cognitive reappraisal in real-life situations is often ineffective [10].

The ACC is a part of a functional system of self-awareness and is implicated across a broad range of emotional processes and behaviors, including contribution to social cognition by estimating emotional facial expressions [169] and how motivated other individuals are and error prediction processing related to costs and benefits during social interactions [170]. ACC dysfunction, perhaps mediated by the inhibitory influence of the amygdala [171], also results in learned helplessness, where an inability to determine the emotional aspect of the difference between the expected reward and outcome results with demotivation and an inability to handle goal-directed tasks, although regions that are engaged in the processing of the task stimuli are even more active [172]. Individually variable degrees of sensitivity to emotional signals, both exteroceptive and interoceptive, also largely depend on the activity of the ACC [173,174]. Although considered to mediate primarily affective functions [175], it is generally accepted that ACC neurons are the main site of integration of attention with visceral, autonomic, and emotional information [174,176,177].

The concept that the “extended amygdala” [178,179,180], where the extended amygdala includes the corticomedial (superficial) complex of amygdala nuclei, sublenticular substantia innominata, the NAc, and the BNST, postulates that the extended amygdala mediates the integration of rewarding (positive) and punishing (aversive) sensory stimuli by translating the motivation generated through the NAc neurons into motor activity [178,181]. There is significant asymmetry in this system in normal individuals because the sensitivity of the cerebral cortex of the frontal lobe to reward stimuli is significantly higher in the left hemisphere than in the right, probably due to the stronger expression of dopaminergic D_2_ receptors [182].

In addition to the amygdala and the OFC, the insula serves a critical role in emotional awareness [58] and is involved in the regulation of emotions, feelings, cognition–emotion integration and social networking [183,184,185,186,187,188]. The insula, due to its strong connection with subcortical and cortical areas that regulate autonomic, physical and emotional information, plays a key role in maintaining homeostasis, and generating emotions and awareness [189,190,191]. Due to its incredible complexity [192] and involvement in evaluative, experiential and expressive aspects of internally generated emotions as a part of the paralimbic cortex, the insula specializes in behaviors that integrate environmental stimuli with the inner milieu [185,193,194].

Patients with damage to insula have a changed decision-making pattern involving risky gains and risky losses, compared to a group of healthy individuals [183,184,195]. Such patients make significantly riskier choices than healthy individuals in a potential gain situation. Therefore, it is suggested that risky decision-making depends on the integrity of the neural circuitry that includes several areas of the brain involved in experiencing and expressing emotions: the insula, amygdala, and vmPFC. Within this neural circuit, the insula is responsible for implicit thinking that makes it easier to face risk and gain in uncertain conditions. The insula is, therefore, probably important in providing an intuitive feeling of correctness when making a decision to avoid or accept risk.

Regarding the abundant connections between the amygdala and numerous subcortical structures and cortical areas, it can be concluded that the amygdala is associated with biological instincts, such as thirst, hunger, and libido, but also with motivation states—the level of arousal, orientation, and response to environmental threats—as well as social, reproductive, and parental behavior [164,165]. All these behaviors are directly related to emotional (affective) states mediated by the amygdala, so there is almost no part of the CNS that is not directly, or at least indirectly, unaffected by the activity of the amygdala.

## 5. Fetal Development of the Amygdala in Human

The primordial amygdala appears about 5.5 weeks after conception. The corticomedial and basolateral groups and the anterior amygdaloid region are the first cell clusters to be identified simultaneously [196,197,198]. The hippocampus is in close contact with the primordial cell clusters of the amygdala, and their neuroanatomical relationship persists until the end of development. Medial forebrain bundle fibers that extend from the tegmentum to reach the olfactory bulb pass near the amygdala. The developmental origin of the amygdala is not entirely understood as to whether it is a diencephalic or telencephalic structure, or a developmentally homogeneous structure. This dates back to Johnston’s first description of the development of the amygdala in 1923, when he hypothesized that the amygdaloid complex consisted of “six or more clusters of cells, some of which represent primitive olfactory areas found in fish and others that are newly formed in terrestrial animals by the process of growth, cell migration and folding of the adjacent piriform cortex” [144]. The amygdala is obviously not a homogeneous structure, for there are similarities in the cytoarchitectural structure with the cerebral cortex and with the basal ganglia. Over time, Johnston’s division has been gradually accepted. Johnston divided the amygdala nuclei into two groups based on embryological and phylogenetic observations. He included the central, medial, and cortical nuclei together with the nucleus of the lateral olfactory stria among the “primitive” cell groups, while he classified the basal and lateral nuclei as phylogenetically younger structures that are formed by cortical ingrowth and cell migration [144]. In his ontogenetic study, Macchi recognized the centromedial complex (the central and medial nuclei), and the basolateral complex (the basal and lateral nuclei) [197]. Macchi also distinguished the anterior amygdaloid area, cortical nucleus, and intralaminar nuclei, but did include the nucleus of the lateral olfactory stria in the amygdala at any stage of development [197]. Crosby and Humphrey divided the amygdala nuclei into a corticomedial and basolateral complex and the anterior amygdaloid region [196,199]. As such, agreement on the amygdala subdivision cannot be reached on the basis of histological and phylogenetic observations alone.

At 6.5 postnatal weeks, cell clusters within the amygdala are larger but still without evident subdivisions, and the first fiber bundles appear [200]. The anterior region is almost equal in length to the rest of the amygdaloid complex; however, its relative size decreases with further development. The axons connecting the amygdala with the preoptic and hypothalamic area pass through this area almost transversely. The ME becomes relatively larger. The basolateral complex, being still a single unit, is continued into the primordial neostriatum, and the primordial primary olfactory (piriform) cerebral cortex is formed. The neuroblasts that constitute the cortical nucleus are not numerous, but rather scattered over the surface of the basolateral complex. The amygdala establishes reciprocal connections with the olfactory tubercle, and the first connections to the epithalamus also form via the striothalamic bundle. At the beginning of the 7th week of gestation, the stria terminalis is already formed, and connections with the cholinergic nuclei in the diencephalon, hippocampus, and diencephalic structures begin to develop [200]. The nuclei of the basolateral group of the amygdala begin to differentiate, with the basal nucleus being especially distinguishable, while the lateral one develops a little later. The NAc, globus pallidus, and medial forebrain bundle can be clearly identified. The putamen suddenly emerges in the 8th gestational week and pushes the amygdala toward the lateral. The central nucleus of the amygdala also differentiates during this period [200].

At the beginning of the fetal period, the development of the cerebral cortex continues, while the differentiation of the main nuclei of the amygdala is completed. The further development of the amygdala in humans, but not in other mammals, causes a change in position, or more specifically, a rotation of structures around the ME. From phylogenetic and ontogenetic perspectives, the ME changes the least, in contrast to the lateral nucleus, which achieves the largest increase in volume and position, being the main afferent structure of the amygdala. Because phylogenetic development shows a tendency to increase the surface area of the cerebral cortex (telencephalization), the evolutionarily increased surface area of the human cerebral cortex is reflected by an increase in the volume of the amygdala nuclei, receiving most inputs from the periphery. At about 12 weeks post-conception, specific, transient ovoid structures develop, especially in the lateral nucleus of the amygdala [200]. Then, the proliferation, migration, and differentiation of nerve cells lead to a rapid increase in the amygdala volume. Around the 20th week of gestation, the transient ovoid structures gradually disappear, and the increase in volume slows down. Repeated increase in volume occurs in the middle fetal stage, probably as a result of the establishment of major connections, primarily frontolimbic [201], but also of efferent projections to subcortical regions of the brain. The amygdala undergoes further changes in the late fetal stage resulting from myelination and other maturation processes, including apoptosis. 

**Figure 11:**
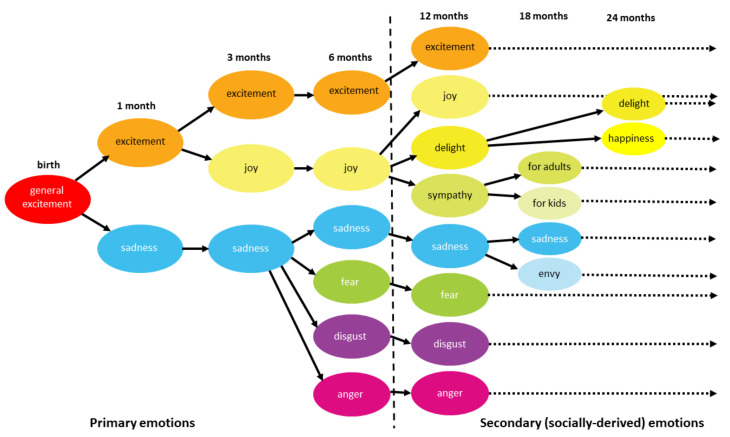
**Ontogenesis of individual primary and secondary emotions in the first two years of life**. According to Banham Bridges (1932) [207].

Further perinatal and postnatal developmental changes of the amygdala are associated with the establishment of structural and functional frameworks and continue to the age of 2 years [202]. The majority of connections are formed or have already been completed at birth, and the pattern of functional development of resting-state default-mode networks follows myelination and maturation [203]. It should be noted that the strong growth of cortical and subcortical gray matter occurs during the first year of life although the cortex matures later [204,205], and the further development of the amygdala, as well as the entire central nervous system, is mainly marked by reorganization, fine-tuning, and reshaping of already-established neural circuits [206]. The ontogenesis of individual primary and secondary emotions in the first two years of life is shown in Figure 11. As stated, the uncinate fasciculus does not finish myelination until about 30 years of age [51].

**Figure 12:**
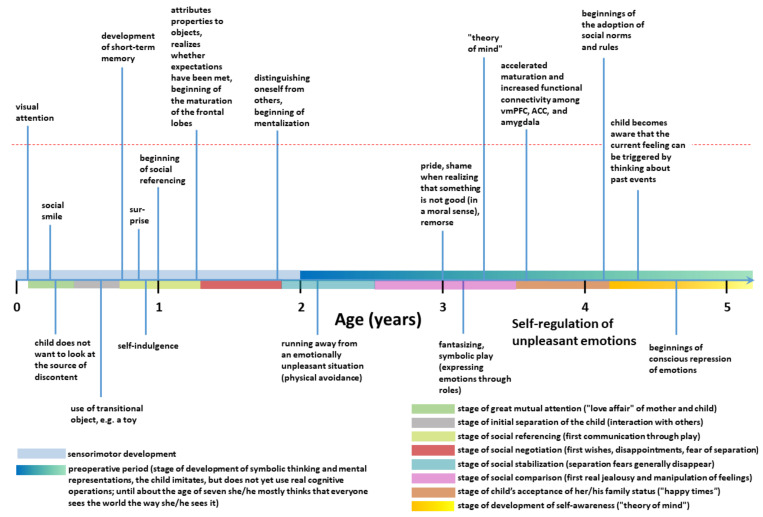
**Schematic representation of the development of select emotional and cognitive abilities in children.** ACC—anterior cingulate cortex; vmPFC—ventromedial prefrontal cortex. The part of the schematic related to the stages of development is made according to Lewis and Granic (2010) [209].

A model of early cognitive development helps to understand when and why certain emotions arise by specifying the cognitive tools that infants or children possess. A schematic representation of the development of selected emotional and cognitive abilities in children is shown in Figure 12. Emotions help children to interpret the world around them and there are different ways (“rationalizations”) by which they cope with emotions with negative valence (strategies of early emotional regulation) that are also dependent on the developed cognitive skills. Although the emotion of fear arises around 6–7 months of age and correlates with the development of amygdalofugal pathways, fear reaches a climax around the age of 18 months and involves the fear of strangers (stranger anxiety) and fear of possible separation from the mother or primary caregiver (separation anxiety). Social referencing refers to children’s ability to understand how they should feel or behave in certain situations [208].

## 6. Damage to the Amygdala and Klüver–Bucy Syndrome

In 1938, Klüver and Bucy described an unusual emotional behavior in monkeys resulting from damage to structures of the medial part of the temporal lobe [210]. The most significant feature of the syndrome was a lack of fear [211], which manifested as a tendency to approach objects that normally cause fear. This deficit is also called “psychic blindness” due to the inability of an individual to attach an emotional value to living beings, events, or objects.

Although monkeys naturally display repulsion, distrust, and a degree of aggression toward strangers, as well as the development of subtle hierarchical relationships with other pack members, those whose amygdala has been partially or completely bilaterally removed are reckless, overfriendly, hypersexual and fearless, not only toward other monkeys but also toward potential predators and unknown/unwanted beings. The behavior is repeated even after having an unpleasant experience, for example, after being bitten by a snake; the monkeys are not afraid to approach them again, and hypersexuality continues toward both sexes even after being beaten by a dominant male.

Because patients with Klüver–Bucy syndrome suffer bilateral damage or removal of the medial part of the temporal lobe, the clinical picture includes deficits related to both hippocampal formation and the amygdala. The main symptom of bilateral structural or functional hippocampectomy is severe global amnesia, or the inability to convert short-term memory into long-term memory. Structural or functional bilateral amygdalectomy causes the following symptoms of Klüver–Bucy syndrome: loss of fear and increased obedience, tameness, non-aggression, oral exploration of objects, hypersexuality, compulsive interest for any visual stimulus (hypermetamorphosis, or utilization behavior), loss of emotionality, visual agnosia (inability to recognize previously known faces and objects) and affective flattening in about half of the cases, and bulimia (hyperphagia with a tendency to eat inappropriate “food”). Klüver–Bucy syndrome can be caused by over 25 different pathological states, ranging from infections, such as shigellosis, to methamphetamine withdrawal [212].

## 7. Emergence of Individual Emotions in the Amygdala

Emotional regulation is extremely important in everyday life, above all in social interactions. The primary role of the amygdala is to facilitate the adaptation of the individual to its environment [104], where emotions with negative valence are associated with an increased activity of the amygdala, whereas emotions with positive valence, such as romantic love, are associated with the deactivation of the amygdala [213]. Dysfunction of the amygdala is primarily associated with disordered emotional regulation of fear and aggression.

### 7.1. Aggression

From a biological perspective, aggression is understood as a survival tool and includes defensive aggression (as in defending the territory and offspring) and predatory aggression (as in competition for food). Other aggressive behaviors that do not meet these criteria are considered pathological [214]. Aggressive behavior is one of the most difficult problems in human society, covering the entire spectrum of behaviors from verbal threats to homicide. As a term, aggression is defined as any behavior that causes harm to others and oneself. Violence is a narrower term within aggression, and means the direct infliction of harm. Such behavior can be impulsive or premeditated. This dichotomous model divides aggression into impulsive aggression, which is a result of an affective reaction to a provocation, where a person cannot resist sudden aggressive instincts “triggered” by an intense emotion of anger, and planned aggression, which does not involve a physiological response [138]. The characteristics of these two main types of aggression and their biological substrates are summarized in Table 1.

The division of aggression types in Table 1 is not absolute because some disorders have characteristics of both impulsive and planned aggression, as may be the case in dissocial (antisocial) personality disorder. Another problem is that most studies of aggression did not use any classification of aggression subtypes. Sometimes, a collection of different characteristics can be found in one individual: antisocial personality disorder (antisocial behavior, impulsiveness, selfishness, emotional insensitivity, lack of empathy and remorse), machiavelism (manipulation, blackmailing, and exploitation of others, lack of morality, violation of social rules for one’s own benefit) and narcissism (a sense of grandiosity and fantasizing about unlimited power, influence, strength and ideal love, complacency and constant obsession with one’s own importance, beauty and uniqueness, demanding excessive admiration, jealousy toward all other people that a narcissistic person perceives as rivals, arrogance)—the so called “dark triad”. Narcissistic personality disorder is about three times more frequent in males than in females (~18% vs. ~6%). At the same time, in those individuals, an internal struggle with lack of self-confidence and failure is often present, and for most of them, the fundamental problem is the incapability to face either the approval or disapproval of other people. Such people do not forgive anyone, often jump from one relationship to another, and usually show aggression only in close relationships (at first glance, they may seem to be successful members of society). A third type of aggression can be added to this dichotomous division: this type occurs under the influence of psychoactive substances, a common example being the sudden onset of aggressiveness in an alcoholic state. 

Traditional understanding suggests that the amygdala releases aggression after PFC decreases its control on the amygdala so that aggressive behavior is further potentiated through “executive centers” in the hypothalamus and sympathetic centers in the spinal cord [214]. Indeed, the amygdala, hypothalamus, and brain stem are thought of as “triggers of aggression” [217]. However, stimulation of medial and basolateral amygdala nuclei in experimental animals is observed to produce aggressive behavior with a range of aggressive behaviors proportional to the degree of activation, whereas decreased activity of the same regions leads to prosocial and submissive behavior [214]. In regard to aggression, it thus seems that there is a functional diversity within the amygdala itself. For instance, stimulation of the ME increases territorial, but decreases predatory, aggression [214]. The ME is also associated with mating and protective behavior toward territory and offspring [219]. On the other hand, stimulation of the CE increases predatory aggression, so the term “independent center of aggression” was coined [214]. Increased activity of the CE is even thought to have a role in pathological aggression associated with reduced emotionality. However, simultaneous activation of the ME and CE is linked to violent behavior [214]. Generally, violent behavior is often the result of several combined factors, particularly increased amygdala activity due to genetic predisposition and an unfavorable environment during early development, that both lead to decreased activity of brain areas responsible for empathic behavior, primarily the vmPFC and OFC [217]. Watching scenes of unjustified violence in normal individuals activates primarily lateral parts of the OFC (processing the punitive stimuli) and insula (empathizing with the victim), whereas the vmPFC is activated only when watching scenes of self-defense [220]. A correlation was observed between reduced amygdala volume and aggressive, violent, and criminal behavior, along with weaker connectivity between the amygdala and vmPFC and OFC, whose activity was decreased [220]. Such persons are incapable of empathy, being unscrupulous and egocentric instead, most often narcissistic and manipulative, incapable of loving and truly caring about someone, and also incapable of experiential learning and feeling ashamed, guilty, embarrassed and regretful. Such behavior together with highly expressed aggressiveness usually begins early in childhood, likely under the influence of genetic and various other factors.

### 7.2. Fear

Fear, the oldest and strongest emotion, played a crucial role in the evolution of vertebrates [10]. While aggression is important for defending territory, protecting offspring and catching prey, fear is essential for facing danger. The amygdala is considered the key structure in preparing an organism to react to danger or engage in a fight-or-flight response [155,156]. Even though it certainly participates in the evaluation of other emotions [146], its role in the detection of fear is primary and evolutionarily the most important [10]. Fear and consequent behaviors are thus, either suppressed or generalized in dangerous situations [80]. Besides its key role in the experience of fear and the fight-or-flight response, the amygdala is crucial in emotional memory [221], processing emotionally charged stimuli from the environment and attributing emotional significance to this information, whether relevant or not [167].

The BLA is considered the key area for the process of fear conditioning [80,129], as demonstrated by LeDoux and others through experiments conducted in rodents [103]. By performing precise neuroanatomical lesions in rats, they revealed that information from sensory systems comes through both the thalamus and the cerebral cortex into the BLA [122]. It has been shown that the BLA decides whether generalization or discrimination will occur during conditioning. The more similar the conditioned stimulus and the context in which each subsequent test was performed at 24-hour intervals, the greater the likelihood of the generalization of fear and the consequent response via the CE [103,109]. BLA activity is increased in anxiety disorders. Its glutamatergic neurons’ excitation generates anxiety, whereas stimulation of GABAergic neurons in the CE reduces it [207]. Moreover, BLA is considered the regulator of social behaviors given that its activity enhances desirable social behaviors and the reward experience, while inhibition of the BLA diminishes it [103]. Furthermore, stimulation of the BLA relieves anxiety and freezing behavior, whereas inhibition produces the opposite effect [109]. 

## 8. The Amygdala and Anxiety Disorders

Anxiety disorders are the most common psychiatric disorders: about 14% of the population meets the criteria for some of these disorders at least once in their lifetime [222]. Although an appropriate response to danger is crucial for survival, it is equally important to distinguish real from false danger [80,103]. If humans were not be able to do so, stimuli signaling danger would cause impulsive fight-or-flight reactions too often. However, if the situation development is correctly predicted and assessed, this control mechanism will prevent unnecessary psychological and physiological reactions [223]. Neuronal circuits involving the amygdala, hippocampus, and PFC are responsible for fear response control, while modulation of amygdala activity mainly depends on the vmPFC.

From an evolutionary perspective, due to the adaptive role in adverse events, the complete absence of anxiety would likely be detrimental. Nevertheless, fear, anxiety, and concern lose their adaptive value in anxiety disorders. In comparison to healthy individuals, anxious persons exhibit two types of changes: the exaggerated experience of fear, sometimes in the complete absence of danger, and subsequent avoidant behavior; and after cessation of danger, persistence of the fear alarm, and the individual behaving as if under constant threat. The current understanding suggests that the basis of anxiety lies in inappropriate regulation of neuronal circuits that supervise emotional and physiologic responses to potential threats [129]. As such, the response to unpleasant stimuli or paired neutral stimuli is either amplified (conditioning) or it subsides. In healthy individuals, there is a balance between these two processes. Pathological anxiety results from excessive arousal of afferent pathways that signalize fear or insufficient activity of descending pathways that inhibit fear-induced behavior [102]. In anxiety disorders, amygdala reactivity is generally increased, not just to threatening situations and stimuli, but also to neutral ones. This finding could explain the experience of severe anxiety in patients with anxiety disorders even in the apparent absence of any real threat. The most differentiating factor between the types of anxiety disorders are the circumstances in which anxiety occurs as well as its intensity (level).

Briefly, a hypersensitive and hyperreactive amygdala, especially its basolateral part, is the common feature of panic disorder, social phobias, and, to a lesser extent, PTSD and generalized anxiety disorder (GAD) [224]. Preclinical studies in experimental animals have shown that such BLA hypersensitivity can be induced by fear conditioning. Due to synaptic changes that mediate associative learning in the BLA, a neutral stimulus is sufficient to elicit a fear response [84]. Increased amygdala activity leads to activation of the hypothalamo–pituitary axis and a subsequent increase in hormone levels (ACTH, adrenaline, cortisol) that produce characteristic somatic symptoms of anxiety. It is thought that increased amygdala activity in anxious people requires greater PFC activity to suppress unpleasant emotions caused by anxiety [129]. However, both the stimuli and the areas of PFC involved greatly differ. For example, it has been shown that the vmPFC suppresses fear by acting upon the BM, while dmPFC exerts a direct effect on intercalated neurons [122]. Both anxiety disorders and depressive disorder share a common feature of increased amygdala activation since in both conditions, the amygdala and entorhinal cortex in the right hemisphere are more active compared to healthy individuals, especially when performing tasks related to the induction of fear or unpleasant emotions in general [225]. Similarly, as in anxiety disorders, greater amygdala activity and reduced PFC activity have been observed in experimental models of depression [226].

### 8.1. Generalized Anxiety Disorder

The key feature characterizing generalized anxiety disorder (GAD) is the inability to differentiate threatening stimuli from neutral ones [224]. Both the volume and activity of the amygdala are increased in people with this disorder, along with amygdala connectivity with other brain areas, especially dlPFC and ACC [227]. The most consistently identified abnormalities in GAD are a hyperactive amygdala and hypoactive PFC [216,220]. Comorbidities with other anxiety disorders and depression are frequently found in patients suffering from GAD, complicating the research and causing inconsistencies in results of different studies [222,228]. 

### 8.2. Social Phobias

A fundamental feature of social phobia is the excessive fear of a negative assessment by other people. Social phobia, as well as other types of (specific) phobias are among the most common anxiety disorders [222]. If protection against an immediate danger is crucial for survival of all vertebrates, a freezing behavior can also be understood as an adaptive mechanism. The CE plays a key role in freezing behavior. A similar process can occur in humans, although instead of defending against predators, activation of such behavior may be triggered by social contexts, such as public performance. In this scenario, additional mechanisms are required to overcome freezing to perform publicly [223]. Individuals suffering from social phobia exhibit increased amygdala reactivity while watching photographs of faces expressing anger or contempt [223]. In these individuals, the amygdala appears to be overly sensitive to frightening stimuli in social situations without altering sensitivity to other contents. Excessive amygdala activity is also associated with decreased activity of the OXT system [134], and the degree of functional connectivity between the amygdala and vmPFC in the left hemisphere is also reduced. Thus, not only the structure of the amygdala, but also the structural integrity of its connections with the vmPFC seem to be disrupted.

### 8.3. Post-Traumatic Stress Disorder

The amygdala is one of the areas in the brain involved in the development of PTSD as the starting point for the process of activation of the hypothalamo–pituitary axis and the cascade of physiological responses to acute stress. An appropriate response to acute stress is a vital adaptive mechanism, but its prolongation causes various biopsychosocial (previously, psychosomatic) disorders. Chronic stress leads to higher expression of CRH/CRF in the CE and BLA, which has an anxiogenic effect [124]. The CRF is considered to be responsible for the anxiogenic effect of different stressors, while OXT has an anti-stress effect [124]. This anxiolytic effect of OXT is mediated by a morphologically distinct subpopulation of astrocytes that express OXT receptors [227]. Furthermore, stress decreases GABAergic activity and also the sensitivity of GABAergic receptors [228]. Reduced activity of GABAergic interneurons automatically leads to overactivity of glutamatergic neurons, and increased excitatory activity in the LA, as already stated, has an anxiogenic effect.

The amygdala mediates both conditioned and unconditioned memory of stressful events, so its activity increases during recollection. Studies using functional magnetic resonance imaging (fMRI) have shown an increase in spontaneous amygdala activity, as well as amygdala activity, when recalling traumatic events [229]. This explains why people suffering from PTSD have hippocampal atrophy without change in amygdala volume. Exposure to chronic stress is thought to lead to impairment of memory dependent on the functional integrity of the hippocampus, whereas memory stored in the amygdala is preserved [230]. Furthermore, BLA levels of brain-derived neurotrophic factor (BDNF) increase under the influence of stress, additionally leading to establishing memory for stressful events, while PFC control over the amygdala is reduced [228]. This helps to explain other characteristics of PTSD, such as impaired memory for facts that are not emotionally significant, while remembering stressful events in detail. 

### 8.4. Panic Disorder

The most defining features of panic disorder are sudden attacks of intense fear with dramatic somatic (sweating, tremor, palpitations, feeling of suffocation, nausea) and cognitive symptoms (fear of death and loss of control). A panic attack can be induced in experimental conditions by infusion of sodium lactate or by inhalation of carbon dioxide [231].

Most people recover from a sporadic panic attack without professional help or treatment, but some develop panic disorders over time if they can no longer clearly distinguish threatening stimuli from neutral ones, and experience inappropriate fear of certain objects, people, or situations. Although very unpleasant, panic attacks are not directly dangerous to health, and can manifest themselves through any form of anxiety disorder (GAD, PTSD, obsessive–compulsive disorder, social phobias) or occur in isolation. In a panic attack, which usually occurs over a short period, in addition to the feeling of great fear (loss of control, alienation from the environment and other people, death), the amygdala strongly activates the ANS, especially its sympathetic part.

Spontaneous panic attacks occur following excessive activation of the amygdala to neutral external stimuli. Individuals suffering from a panic disorder have reduced volumes of the right LA and BLA and therefore, the volume of the right amygdala is significantly reduced, too [162]. The LA recognizes sensory stimuli and the BLA detects potential threats and forwards this information to the CE, resulting in the aforementioned somatic symptoms of sympathetic activation. The misperception of danger likely precedes a panic attack, which is especially true for the misinterpretation of bodily sensations [232]. Furthermore, people with panic disorders over-process images of frightened faces compared to healthy controls, which is associated with greater amygdala activation [233]. Preclinical models indicate a disturbed balance between excitation and inhibition in the BLA and CE [231,232]. A reduced density of GABAergic neurons in the BLA is, in fact, correlated to an increase in the intensity of fear [231]. The amygdala represents the main hub of the fear network in a panic disorder, which also includes the thalamus, hippocampus, hypothalamus, PAG, and brainstem [232] for which individuals suffering from a panic disorder have a lowered threshold for activating.

## 9. The Role of the Amygdala in Consumption and Negative Effects of Alcohol

The consumption of alcohol induces a change in emotional state in terms of relaxation and euphoria, in addition to relieving stress and anxiety [234,235]. Nonetheless, alcohol shows a broad spectrum of effects, ranging from altruistic to extremely aggressive behavior [236,237,238].

Alcohol disrupts the balance between inhibitory and excitatory neurotransmission in the amygdala. GABAergic transmission in the BLA and CE is enhanced under the influence of alcohol, while glutamatergic transmission is reduced in the same regions [124]. The acute action of alcohol is anxiolytic, sedative and positively reinforcing [124]. The anxiolytic effect of alcohol appears to be achieved primarily through the action of ethanol on the amygdala, while the euphoric effect is achieved by stimulation of the NAc [239]. Like other sedatives, alcohol mostly acts through GABA receptors. In small amounts, it enhances the action of GABA-A and GABA-B receptors, and in larger quantities, the release of dopamine activates serotonergic 5-HT_3_ receptors and blocks NMDA receptors. In addition to sedation, the short-term effects of alcohol include loss of inhibition, decreased anxiety, and impaired motor coordination [240,241,242].

However, chronic alcohol consumption leads to hyperexcitability of glutamatergic systems, most pronounced in the development of tolerance in which decreased effects of alcohol in the NAc and amygdala occur [239], and in withdrawal syndrome. Individuals with increased excitability of BLA pyramidal neurons are more anxious and have a greater tendency to consume alcohol [239]. Alcohol reduces amygdala activation when observing faces expressing fear [239]. Early life stress and chronic stress later in life [243] lead to increased excitability of the BLA, and alcohol can reverse that effect. However, the link between alcohol and stress seems to be bidirectional. Due to the development of tolerance, the anxiolytic effect of alcohol gradually weakens, while amygdala excitability [239] and addiction increase [244]. Lack of alcohol leads to dysphoria and craving, further supporting the addiction. In a state of developed dependence, the reward system is active but a normal reward no longer activates it, as predicted by the incentive–sensitization theory [245] and confirmed by functional imaging studies [246].

In addition, alcohol impairs the processing of emotions within the amygdala, so that a person under influence can misinterpret another person’s behavior as threatening. Alcohol disrupts the connections between the PFC and amygdala, leading to reduced control over executive functions, which includes consideration of consequences, control of behavior and cognitive assessment of the self and one’s social relationships [247,248].

## 10. The Influence of the Amygdala on the Brain Reward System

Unpleasant emotions, such as fear, have great and overriding adaptive value. However, the ability to feel satisfaction for any subjective success is also necessary for a long and healthy life [249]. Pleasant emotions are a catalyst for success because they enable and encourage problem solving, cognitive flexibility, social cooperation and the achievement of goals [249]. A high level of positive emotion is associated with a greater degree of optimism, self-confidence, and efficiency, as well as better regulation of emotions and self-well-being [250]. Although moral and social norms impose altruism and reciprocal relations, the goal of most is always the greatest possible subjective well-being with the lowest possible price/effort. From that perspective, the feeling of reward (pleasure) can be seen as “the greatest trick of evolution” [251]. Although this trick serves to motivate the individual to achieve the greatest possible ability to survive and reproduce, it can also be a source of affective disorders, addiction and psychopathology, especially in modern, wealthy societies [251].

A reward is any object, event, stimulus, situation or activity that induces pleasant emotions, leads to behavior aimed at approaching the source of the reward and results in positive reinforcement that operates under the principle of maximization in decision-making, such as to maximize pleasure/benefit and minimize pain/discomfort with least cost [252]. In psychological terms, the brain’s reward system consists of a number of components of which three are the most important: (1) liking—a fundamental reaction to a stimulating hedonic stimulus; there is a general consensus that the opioid system is the most important for the process of liking (e.g., the injection of opioids will induce both liking and desire); (2) wanting (desire)—a reflection of motivation toward some incentive sensory stimulus; the process of motivation is mediated primarily by the mesocortical dopaminergic system (see below); and (3) learning—most often in the form of classical or instrumental conditioning, or cognitive representations [253].

Each of these components can be further divided into an explicit and implicit subcomponent. We experience explicit processes consciously, while we cannot always be aware of the implicit ones (e.g., we may like or dislike someone or something, but we are not aware of it). Consequently, the explicit liking consists of conscious hedonic feelings, while the implicit “liking” includes all affective reactions whether we can measure them objectively or not. Unlike liking, wanting (desire) does not contain any hedonic (sensory) pleasure, so it can neither increase nor decrease it. Although part of the larger whole of the brain’s reward system, wanting (desire) is thought to be largely dependent on decision-making when faced with multiple potential goals at the same time [254]. The explicit wanting subcomponent consists of subjective, goal-oriented plans and all known or imagined stimulating cognitive representations that we know or assume, or have some kind of cause-and-effect understanding of how to achieve them. In addition, for their realization we expect that they will be directly pleasant. Implicit “wanting” means all possible rewards and their indications when this motivational value is assigned to them by the dopaminergic mesolimbic system during unconscious processing (see below). Thus, implicit rewards may suddenly become “motivational magnets”. For example, due to some stimulating feature, the animal may be motivated to eat an inedible object or a cocaine addict may collect crack crystals from the floor even though she/he knows that it is actually just crystalline sugar [255]. Due to the fact that they do not represent common, conscious desires whose presumed outcome is always known, such implicit “desires” derived from mesolimbic activation are put in quotation marks [255]. Explicit learning refers to all types of learning based on conscious expectation of a reward, as well as those in which we understand the cause-and-effect nature of outcomes, while implicit learning includes all forms of associative learning, especially conditioning and reinforcement, which do not require awareness or attention. Although the above division is made for didactic reasons and simplification, no component of learning can be separated from the influence of emotions operationalized through projections of the amygdala onto the different parts of the reward system.

**Figure 13:**
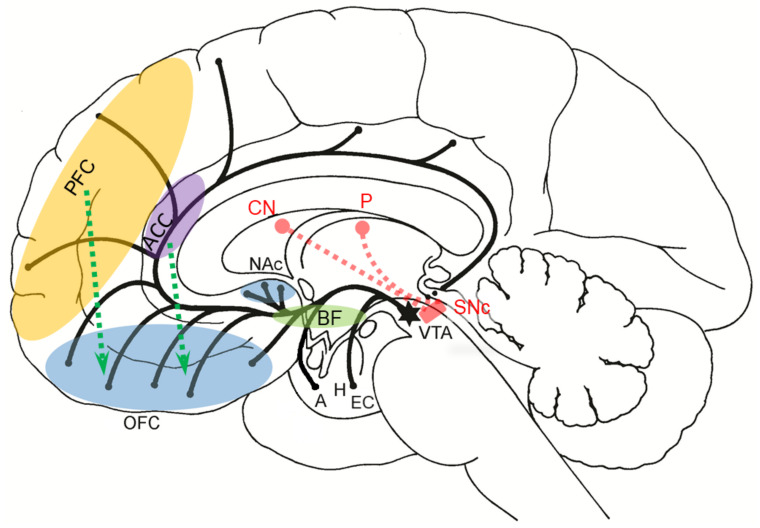
**Schematic representation of dopaminergic projections that make up the brain reward system.** The projections originate from the neurons of the ventral tegmental area (VTA, black star) and go to the ventral striatum (ventral pallidum), especially the nucleus accumbens septi (NAc, small blue ellipse, mesolimbic pathway), orbitofrontal cortex (OFC, large blue ellipse) and prefrontal cortex (PFC, yellow ellipse, mesocortical pathway), anterior cingulate cortex (ACC, purple ellipse) and mediobasal telencephalon (basal forebrain, BF, green ellipse), entorhinal cortex (EC), hippocampus (H) and amygdala (A). The release of dopamine from projecting VTA neurons in other parts of the CNS, especially the hippocampus (H) and the amygdaloid nucleus (A) is associated with the memory of (otherwise neutral) individual stimuli/objects/events present during rewarding, which gives them motivational importance [256,257,258]. It is thought that dopaminergic projections from the substantia nigra, pars compacta (SNc, red rectangle) to the dorsal striatum, i.e., caudate nucleus (CN) and putamen (P) also transmit information that associate salient sensory stimuli with reward and reward prediction error, but in this context they are associated with the dopaminergic “tone” necessary to perform conscious motor movements and to reprogram motor patterns that will facilitate obtaining the same reward in the future [259]. Green dashed arrows represent projections of the PFC and ACC in the OFC. These projections are thought to exert cognitive (top-down) control over glutamatergic and GABAergic interactions in the OFC, a key region responsible for making behavioral choices, such as emotional go/no-go decisions. Schematic modified from Šešo-Šimić et al., 2010 [169].

Neuroanatomical, electrophysiological and neuropharmacological experiments conducted until the mid-1980s revealed that four groups of interventions applied in experimental animals could lead to reward, including injection of amphetamines into the NAc, injection of morphine into the VTA, electrical stimulation of the VTA, and electrical stimulation of the medial forebrain bundle (MFB). Based on these and other findings, main elements of the neural circuits that make up the brain’s reward system have been defined (Figure 13).

The most important reward pathway in the brain is the mesocorticolimbic dopaminergic system, the backbone of which is composed of the VTA, NAc and OFC. Midbrain dopaminergic neurons in the VTA play a key role in reward-dependent motivation and behavior and are controlled by projections from the rostromedial tegmental nucleus (RMTg) and the dorsal raphe nucleus (DRN). Through projections from the VTA into different parts of the CNS, dopamine attaches motivational valence to the processed contents in order to create a sense of current (projections to NAc) or future reward (projections to the PFC), adjusts the value of the stimuli in light of the new experience/context, creating a sense of satisfaction associated with the stimulus or its cues (projections in the NAc and ventral striatum), and supports the consolidation of associative conditioning (projections to the amygdala) and episodic memories (projections to the hippocampus). Due to the secretion of higher amounts of dopamine in the striatum, all naturally rewarding activities lead to increased motor activity: when happy, we jump; and when sad, we stay, helplessly, for a long time in the same place. Dopaminergic projections from the SNc into the dorsal striatum (caudate nucleus, CN, and putamen, P) also serve to reprogram motor patterns that will facilitate the realization of the same award in the future. In addition to motivation (“wanting”, desire), through the amount of dopamine secreted in the NAc, dopaminergic projections from the VTA also encode an error between the predicted and realized level of reward, due to which the subjective value of the reward is constantly changing [260,261,262].

Addictive drugs are initially rewarding, mediated by the NAc, septum and other areas of the ventral striatum, but also reinforcing, mediated by the neurons in the VTA. Repeated reinforcement results in the sensitization of NAc neurons and the creation of a strong desire (“wanting”) to re-take the drug despite increasing dislike and negative consequences: mental and physical dependence, tolerance, and craving (incentive–sensitization theory) [245,263,264]. The theory has a broader impact not only on the explanation of addiction and drug addiction, but is also applicable to the explanation of all other addictive and compulsive behaviors, such as gambling, shopping (binge shopping), binge drinking, overeating (binge eating), excessive need for sports, sexual activity and the use of pornographic content, where only desire (“wanting”) is expressed and liking is often lacking.

The mesocorticolimbic pathway conveys information relevant to associating perceptually incentive sensory stimuli with the reward as well as reward prediction error, that is, the relationship between the rewarding stimulus and expectation [259]. For example, if in a restaurant we get better food than we expected, it will increase our predictions that the food in that restaurant will be good, so we will probably come again [260]. Moreover, the error of predicting a reward that codes for the subjective value of any reward through dopamine secretion in the NAc has much deeper and more far-reaching effects, namely, when we analyze the positive errors of the expected reward, such as rewards that are higher than expected, our expectations for future rewards also increase. In the case that the first subsequent prize deviates less than the predicted error, it will also produce a less positive error of the expected prize. Therefore, we will need ever greater rewards in order to achieve the same error of predicting the reward and the same degree of satisfaction [260]. Consequently, we will constantly seek an ever greater reward (pleasure). Such maximization of the reward is certainly useful in evolutionary terms because animals and humans do what they enjoy since pleasure is a “side effect” of achieving some evolutionary goal, such as feeding or reproduction. The feeling of comfort through evolution is set up in such a way that the pleasure cannot last forever because we would no longer think of survival and reproduction. Therefore, the anticipation of pleasure is extremely strong, and the pleasure itself is only short-lived. Thus, the search for an ever-increasing degree of comfort also has undesirable “side effects” for everyday life, such as the creation of a constant desire for increased economic consumption beyond the required existential minimum. Such a distorted perception, namely the disproportion between desires and possibilities, due to the inability of economically rational control of making intertemporal choices, can lead to emotional crises, various affective and eating disorders and psychopathology [260,261,262,263,264,265].

**Figure 14:**
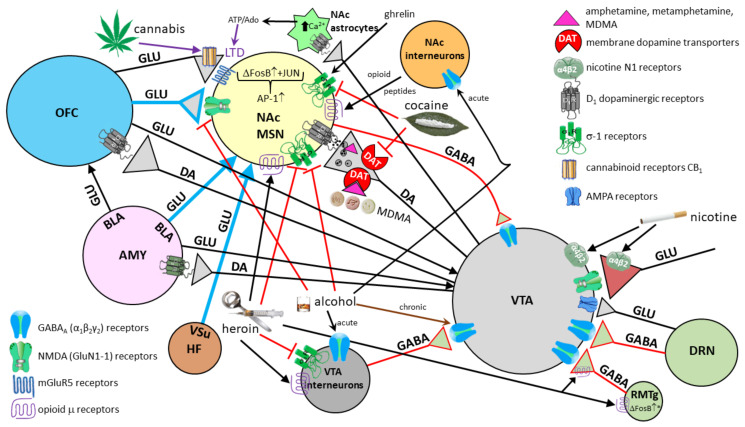
Schematic drawing of the main sites and mechanisms of action of some common addictive drugs on the brain reward system: reward learning and motivation are strongly influenced by the amygdala. Thick blue arrows from OFC, AMY, and HF to NAc convey contextual information associated with the addictive substance and contribute to relapse. Although many addictive substances directly stimulate the release of dopamine from neurons in the VTA that are projected into the NAc, it must not be forgotten that the same effect (activation of VTA) with drug-related stimuli can be achieved indirectly through projections from the amygdala to the PFC and then from PFC to VTA [265]. In a state of developed dependence, the reward system is active, but the usual (normal) reward can no longer activate it. This state of motivational toxicity is expressed in hardened addicts. It is manifested by a lack of interest in career, social and sexual relations, financial status and increased engagement in the procurement and consumption of drugs. The diagram does not show the efferent projections of NAc that go to the basal ganglia and ventral pallidum. Neurons of the ventral pallidum are projecting through the mediodorsal nucleus of the thalamus into the PFC and striatum, and additional projections go into the RMTg, the compact part of the substantia nigra (SNc), and the reticular formation of the pons. Not shown are glutamatergic projections from the thalamus and ACC into Nac, as well as projections of NAc and ventral pallidum into the lateral hypothalamus. AMPA—α-amino-3-hydroxy-5-methyl-4-isoxazolepropionic acid; AMY—amygdala; AP-1—transcription factor activating protein 1; ATP/Ado—adenosine triphosphate/adenosine; BLA—basolateral nucleus of amigdala; DA—dopamine; DRN—dorsal raphe nucleus; ΔFosB & JUN—truncated member of the Fos family of transcription factors and JUN protein (ΔFosB↑* in RMTg applies only to psychostimulants); GABA—γ-aminobutyric acid; GLU—glutamate; HF—hippocampal formation; LTD—long-term depression; MDMA—3,4-methylenedioxymethamphetamine (ecstasy); NAc MSN—medium spiny neurons in nucleus accumbens septi; OFC—orbitofrontal cortex; RMTg—rostromedial tegmental nucleus; VSu—ventral subiculum; VTA—ventral tegmental area. See text for details.

Mental and physical dependence do not have to be the same for each addictive substance. For example, due to anxiety, anhedonia, depression and suicidal thoughts, psychological dependence on cocaine in abstinence is usually much higher than physical dependence, while in heroin addiction, the opposite is true: physical symptoms of withdrawal syndrome, such as vomiting, diarrhea, muscle cramps, sweating, tremor and insomnia, are more severe than those that are psychological. The term tolerance refers to the reduced effect after repeated intake of the same dose; to achieve the same effect, it is necessary to constantly increase the dose of the addictive agent. Upon restraint, the reward system does not return to its initial state, because sensitization occurs—a process opposite to tolerance [265]. Sensitization is thought to occur due to the accumulation of the transcription factor ΔFosB in the NAc, which activates numerous, still insufficiently known, genes and signaling pathways, including those important for synaptic plasticity, long-term potentiation and consolidation leading to morphological restructuring of dendritic spines as one of the most important cellular substrates of long-term memories associated with addictive substances [266]. The consequence of sensitization of neurons in the NAc is craving, a drug seeking behavior. A simplified scheme of the main sites and mechanisms of action of some common drugs of dependence on the brain’s reward system and the modulating role of the amygdala according to recent research findings are given in Figure 14.

In the case of failure in achieving the planned goals and the expected reward, especially in situations of chronic stress when free cortisol levels become significantly elevated, a person will unconsciously activate the brain reward system in some other, usually well-known and direct way. Such poor intertemporal choices (which favor short-term gain rather than long-term success), as well as the inability to refrain from immediate comfort to achieve a later, more rewarding goal, are associated with poorer emotion regulation. Additionally, although many people experiment with various addictions and drugs, from legal ones, such as coffee and cigarettes, to illegal ones, a relatively small number of them develop a real and complete addiction. Both of these individual differences may be associated with a pattern of attachment at an early age [169]. Numerous experimental results support this conclusion. For example, one experimental model showed that rats that were maternally separated and those that were non-handled for the first 14 days after birth were later hyperactive when moved to new environment, and also showed significantly higher sensitivity to cocaine and amphetamine-induced locomotor activity [267]. These animals, compared to controls, had a significantly higher increase in dopamine in NAc after a mild stress (such as tail-pinch test) [267]. This result confirms that lack of care and attachment relationships during the early postnatal period leads to profound and long-term changes in emotional development with consequent increased reactivity of the mesocorticolimbic dopaminergic system to stress and addictive substances. Due to the slower maturation of the OFC, such children will exhibit disinhibition syndrome more often in later childhood, will not be able to calm down easily even in mildly stressful events and communicate their negative emotions with the primary caregiver in the way that their peers do.

Finally, it is worth emphasizing that both very stressful situations (e.g., crises after natural disasters) and highly rewarding atmospheres (e.g., shopping malls) activate the mesocorticolimbic dopamine system. In such situations, it is more likely that we will judge an incentive stimulus as “desirable” and, for example, buy something that we do not particularly like. Experimental data of optogenetically controlled dopamine release from VTA neurons in the NAc confirmed that a (controlled) increase in the concentration of secreted dopamine in NAc prior to reward increases the sensitivity of a conditioned rat to the price (the required number of lever pressures per food pellet) to be paid for that reward, whereas an increase in dopamine release in NAc after the prize is given makes the animals less sensitive to the price [268,269].

## 11. Short Description of Clinical Cases Presenting with Disturbed Emotional Experience and Behavior

Probably the most known cases of disturbed emotional experience and behavior in the history of neuroscience is the case of Phineas Gage, a man whose injuries unequivocally showed that damage to the frontal lobe affects personality, behavior, and emotional experience [270]. A detailed analysis of Gage’s skull using structural MRI showed that the iron bar severely damaged the frontal lobe of both hemispheres, with the most pronounced damage to the left ventromedial prefrontal cortex, an area crucial for decision- making and emotional regulation [271]. A re-analysis of the Phineas Gage case confirmed the assumption that the PFC, especially its ventromedial part, is associated with emotional regulation. This conclusion is strongly supported by the reciprocal association of the vmPFC with subcortical structures, primarily the hypothalamus and amygdaloid nucleus, which control and regulate fundamental instinctive behaviors aimed at survival and reproduction (hunger, thirst, fear, escape, aggression, libido), the autonomic and endocrine systems, emotion processing and social cognition [272]. Recent research indicates Gage’s extensive damage to the white matter of the frontal lobe as well as the white matter of the anterior parts of temporal lobes and amygdala, as evidenced using a virtual tractogram (diffusion tensor imaging, DTI) of his traumatic brain injury [273]. Unfortunately, insufficient details on Gage before and after the accident have been preserved to allow more precise pathological–clinical correlations and unambiguous conclusions to be drawn about the effects of his injury on subsequent behavior.

Patient S.M. had very pronounced bilateral amygdala damage caused by the rare autosomal recessive Urbach–Wiethe disease, resulting from a mutation in the extracellular matrix protein 1 gene. Due to the mutation of this gene, numerous pathological changes occur, the most pronounced being the deposition of hyaline material in the patient’s skin and bilateral calcification of the amygdala and periamygdaloid gyrus in 50–75% of patients, usually starting at the age of 10 [274]. The general intellectual and other basic perceptual and cognitive abilities of patient S. M. were within normal values at the time of admission to the hospital. It is therefore not surprising that between the ages of 10 and 20, she did not notice that she cannot feel fear, and she was brought to the hospital at the age of 20 due to symptoms of epilepsy. Severe amygdala atrophy was revealed first by computed tomography (CT) and thereafter by MRI, whereas the adjacent white matter showed only minimal damage. During neuropsychological testing, S. M. showed highly specialized impairment associated with the emotion of fear [275]. For example, she did not show a conditioned electrodermal response to fear, had difficulty recognizing facial expressions showing fear (but could recognize facial expressions of other emotions), and did not feel fear (while experiencing other emotions normally). However, S. M. experienced a panic attack after inhaling carbon dioxide (which usually causes a feeling of suffocation), indicating that the panic state resulting from suffocation does not require amygdala activation. She was also prone to fear conditioning in certain situations—for example, she refused to seek help of a dentist because of the pain she had experienced at the dentist’s previously [61]. Finally, S. M. did not have an inability to understand the concept of fear, e.g., she could clearly describe situations that could evoke fear, as well as sounds in voice recordings that reflected fear, indicating that the conceptual knowledge of emotions is largely separated from the emotional states themselves. Therefore, thinking about emotions (e.g., the use of terms and words associated with emotions), conscious experience of emotion and emotional state are three different phenomena.

The case of 14-year-old boy B. W. with a congenital focal malformation of the left vmPFC was published by Boes et al., in 2011 [276]. At the age of 6, his parents noticed that the boy had become disobedient and defiant both at school and at home: there were minor incidents of theft (e.g., stealing cookies, which he then sold), lying, aggression, anger, swearing, disobedience, and carrying a pocket knife to school. At the age of 7–9, this behavior worsened, and since punishing the boy had no effect, he continued to study from home. Despite behavioral problems and a lack of motivation, B. W. showed an enviable level of intelligence. At the age of 11, he was admitted to the emergency department due to feelings of hopelessness, worthlessness, and suicidal ideation that lasted for two months with worsening of the aforementioned symptoms—the boy was even more aggressive, destructive, non-empathetic, impulsive, hyperactive and hypersexual, even though he had not yet reached puberty (he constantly watched pornographic websites and demanded peers to undress in front of him). Although he could not plan well, he still tried to manipulate other people with the sole purpose of satisfying his personal needs, just like an inveterate psychopath. He would get angry and have uncontrolled outbursts of anger if others prevented him from accomplishing anything he intended. He showed deep disrespect for any authority and disturbed moral judgment. He used a lighter to set fire to the house where he lived and several times to the church he went to with his parents. He was arrested for attempted burglary. He lied and stole without remorse. He threatened his mother with a knife. Because his father restrained him from hurting his brothers and sisters, he hit him hard in the head with a wrench; according to his father, he did so coldly, “without any emotion”. Unlike previous MRI images taken at the ages of 4 and 9 years on a 1.5 T MRI device, only now, at the age of 13, has a 3 T MRI scan been taken, and this finally explained his clinical picture of a complex partial epilepsy and behavioral disorder. The main findings were a focally thickened cerebral cortex, loss of a clear boundary between gray and white matter, and enhanced white matter signal of the gyrus rectus, i.e., the vmPFC and adjacent areas (in T2 and fluid attenuated inversion recovery (FLAIR) sequences; these two methods are best for detecting changes in white matter). The signal could not be improved by the contrast, and the hyperintensity of the white matter of the gyrus rectus spread toward the frontal horn of the left lateral cerebral ventricle, suggesting a possible Taylor-type focal cortical dysplasia radial migration disorder, but this was not confirmed by postoperative histological analysis. The MRI showed that the malformation affected parts of Brodmann’s areas 11, 12, 25 and 32. Extensive preoperative mapping of the entire vmPFC revealed small clusters of dysplastic neurons in the left amygdaloid nucleus and the adjacent cerebral cortex of the anterior medial and lateral temporal cortex, which was confirmed by neuropathological analysis after resection. Similarly to other people with left vmPFC damage, B.W. could not pass the Iowa gambling task, i.e., learning from which decks it is good to take cards [49]. The behavioral and neuropsychological profile of B.W. is consistent with previously described cases of focal vmPFC damage and the amygdala disconnected from the frontal input. Therefore, it comes as no surprise that, just like most other patients with pathological changes or injury to the vmPFC, B. W. had relatively normal performance on standard neuropsychological tests.

Other comparable cases of disturbed behavior in relation to emotional experience include the following: the case of patient B., who suffered from bilateral damage mainly of the insula due to *Herpes simplex* infection [183]; patient Roger, who suffered from bilateral damage to insula, ACC, and amygdala also due to *Herpes simplex* encephalitis [184]; and patient A.P., who, similar to S.M., had bilateral calcification of the amygdala due to Urbach–Wiethe disease [277]. The neuropathological findings and altered behaviors of these six cases are summarized in Table 2.

Patient B. is important, as this case showed that despite bilateral destruction of the insula, which caused the olfactory and taste changes, he had normal emotional reactions and feelings. Thus, the authors concluded that it is the subcortical level that ensures basic feeling states, while the cortical level of emotion processing probably largely relates feeling states to cognitive processes, such as decision-making and imagination [183]. Similar to patient B, patient Roger also had bilateral herpes simplex damage to the insula, ACC, and amygdala [184]. The patient’s cognitive abilities were within normal ranges, including speech, language, attention, working memory, and metacognition. His major deficits included global amnesia, anosmia, and ageusia, while his pain experience was not impaired (but sometimes even intensified), confirming that the insula, ACC, and amygdala (structures of a putative “pain matrix” that has been suggested to reflect the affective dimension of pain) are not necessary for feeling the suffering inherent to pain. Roger’s heightened degree of pain affect actually suggests that these regions may be more important for the regulation of pain rather than providing substrate for pain’s conscious experience [184].

Regardless of the fact that in patients such as S.M. [279,280,281] and A.P. [277], due to calcification of the amygdala, the dominant finding was a loss of fear, it should be emphasized once again that the amygdala (especially the left amygdala) is not only involved in generating and processing the emotion of fear, but also with other types of emotional signals, including the generation of loss aversion, including monetary loss aversion, by inhibiting actions with potentially deleterious outcomes [278]. When tested, patients A.P., A.M., and B.G. showed a greater tendency than the controls to rate occluded-face stimuli (occluded-face stimuli contain less information than whole-face stimuli) as more approachable than whole faces, which suggests that the amygdala’s role in approach behavior extends beyond responses to specific stimuli [282]. The electrophysiological and fMRI studies demonstrated that individuals with unilateral or bilateral amygdala injuries have also significantly impaired recognition of a number of different social emotions, such as guilt and adoration, compared to control groups [4,283,284]. The fact that these individuals are more likely to have significantly impaired recognition of social rather than fundamental emotions further confirms that the amygdala also specializes in processing stimuli with complex social meaning and significance.

## 12. The Role of the Amygdala in Sensation Seeking, Psychosis, Major Depression and Other Psychiatric Disorders

Distinct morphological and functional features of the amygdala have been reported across psychiatric disorders. The amygdala plays a key role in both emotional processing and stress response; alterations in amygdala neural activation on emotional tasks were reported in patients with disorders associated with stress and disturbed emotional perception, such as affective disorders. However, amygdala reactivity on specific cues was not uniform across the affective disorders spectrum, given the different amygdala activation patterns during emotion processing in unipolar depression and bipolar disorder. Of note, the majority of fMRI studies showed greater amygdala activation on negative emotional stimuli in unipolar depression than in bipolar disorder, while the opposite was reported for positive stimuli [285]. While increased amygdala activation was observed in patients with bipolar disorder across all illness phases, similar findings were also observed during attention tasks that had no emotional components, suggesting the additional role of the amygdala in cognition [286]. A recent meta-analysis reported smaller amygdala volumes in participants with major depressive disorder (MDD) compared to healthy controls, although greater differences between groups were observed for hippocampal volume [287]. Interestingly, amygdala volumes in bipolar patients did not differ from healthy controls [288].

Negative emotions that are induced by telling a subject that a painful stimulation will be delivered shortly may result in either amplification of pain if a mild pain stimulus is delivered (hyperalgesia) or in the perception of pain when a tactile stimulus is applied (allodynia) [279]. In other words, anxiety about pain activates brain circuits that may increase or decrease the feeling of pain. Using this paradigm, neuroimaging studies in patients with MDD compared with healthy controls showed significantly lateralized perception of pain in depressed patients, as thermal pain tolerance and electrical pain tolerance were significantly increased on the right hand side [280], and impaired ability to modulate pain experience in MDD patients, due to increased emotional reactivity during the anticipation of pain. Subjects with MDD compared with healthy controls showed increased activation in the right anterior insula, dorsal part of the ACC, and right amygdala during anticipation of painful, relative to nonpainful, stimuli, increased activation in the right amygdala and decreased activation in the PAG, rostral ACC and PFC during painful stimulation relative to nonpainful stimulation, and greater activation in the right amygdala during anticipation of pain, which was associated with greater levels of perceived helplessness [281].

A recent metaanalysis comprising 1141 patients and 1242 healthy controls in 54 studies showed that both young and adult patients with MDD showed abnormal neural activities in the ACC, insula, superior and middle temporal gyrus, and occipital cortex during emotional processing. However, hyperactivities in the superior and mid frontal gyrus, amygdala, and hippocampus were observed only in adult patients, while hyperactivity in the striatum was only found in young patients compared to the controls [289]. Apart from the fact that both young and adult patients with MDD have the negative processing bias during emotional processing, these findings suggest that adult patients with MDD are more subject to impaired appraisal and emotional reactivity, while young patients with MDD are more prone to an impaired perception process [289]. After comparing 313 MDD patients with 283 healthy controls, another metaanalysis of the resting-state functional activity in medication-naïve patients with their first episode of MDD revealed that MDD patients had significant and robust resting-state hyperactivity, mainly in the left amygdala and the left hippocampus [290]. These results confirmed the earlier notion that the left hyperactive amygdala in depression affects both the onset and maintenance of emotional dysfunction by eliciting dysfunctional negative biases at automatic stages of affective information processing [291].

Real-time fMRI coupled with neurofeedback allows a person to see and regulate the localized hemodynamic signal from his or her own brain. Using this method, an applied neurofeedback training was given to healthy and depressed individuals with the amygdala as the neurofeedback target to increase the hemodynamic response during positive autobiographical memory recall. The initial results of this approach are encouraging and suggest its clinical potential in alleviating symptoms of depression [292], especially stress-induced depression [293].

In sharp contrast to MDD, patients with schizophrenia, even in the early phase, had smaller amygdala volumes relative to both healthy groups and bipolar patients [288]. Patients with schizophrenia had also decreased structural connectivity between the amygdala and orbitofrontal cortex and abnormal resting-state functional connectivity with the medial prefrontal cortices [288]. Such findings may be related to specific symptoms of schizophrenia. For example, increased amygdala activity may have a role in distress and the perception of threat, related to auditory hallucinations [294]. There are also important differences in the nature of motivational deficits associated with psychosis vs. depression. Namely, depressive individuals, particularly those who experience anhedonia, have the presence of impairments in in-the-moment hedonics (“liking”), and such deficits may propagate forward to impairments in other constructs that are dependent on reward responses, such as anticipation, learning, effort, and action selection, which could reflect alterations in dopaminergic and opioid signaling in the striatum related to depression or specifically to anhedonia in depressed people [295]. In contrast, there is relatively intact in-the-moment hedonic processing in psychosis, but there are impairments in other components involved in translating reward to action selection. In particular, psychotic individuals exhibit altered reward prediction and associated striatal and prefrontal activation, impaired reward learning, and impaired reward-modulated action selection [295].

Individuals with sensation-seeking traits have generally higher thresholds for threat detection, which may arise from amygdala—inferior frontal gyrus interaction. Inferior frontal gyrus suppresses amygdala activity, resulting in feeling less fear, which may result in reckless behavior of drug abuse [296]. Sensation seeking is associated with an initial blunted amygdala response [297], which may result in pursuing more stimulating rewards, using risky and reckless behavior. Sensation (novelty) seeking is defined as the motivation to seek out novel, complex, and arousing experiences and is one of the three main independent dimensions of temperament (the other two being reward dependence and harm avoidance) and one of the four main independent dimentions of impulsivity (the other three being lack of premeditation, lack of persistence, and urgency) [298]. Impulsivity is considered a major endophenotype associated with disorders of behavioral control, such as substance use and pathological gambling, as well as co-morbid neuropsychiatric disorders, such as bipolar disorder and borderline personality disorder [299].

Adolescents endorse greater sensation- and novelty-seeking motivation and reduced behavioral markers of anxiety than adults (with the peak of sensation seeking coming and going earlier in females than in males). From an evolutionary perspective, orientation toward novelty seeking and risky actions could represent an advantageous mode of interacting with the environment during adolescence, given the heightened demands on adolescents to find novel territories, mates, and resources [300]. Sensation seeking is closely related to the extent to which adolescents utilize emotionally relevant information in decision-making, e.g., concerning the gain and loss of territories, mates, and resources.

Using the Iowa Gambling Task to quantify approach vs. avoidance-based decision-making in children, adolescents, and young adults, Cauffman and colleagues (2010) [301] found that levels of approach toward potential reward took on a curvilinear function, with the maximal sensitivity to positive feedback and risky choices (including risky [unprotected] sexual behavior) occurring during the adolescent years (peaks in late adolescence around ages 18–20; in contrast, use of negative feedback to avoid negative outcomes strengthen with age in a linear manner, not showing full maturity until the adult years). This age trend of sensation seeking has been replicated across many cultures [302,303] and confirms the conventional wisdom saying that people become more cautious and conservative with age. However, adolescents do not reveal these tendencies in all situations, but only in the arousing, thrilling contexts [304,305], when they tend to disregard information about the odds of gain and loss and report greater reliance on “gut-level” and “excitement” cues to shape their choices, ultimately impairing their performance. The social context has been shown also to propel adolescents’ decision-making in the direction of risk. Adolescents are more likely to make dangerous moves while driving in the presence of peers [306] and are more prone to deviant behavior when with others than when alone [307]. It still needs to be clarified which of the proposed potential mechanisms predominantly underlie peer influence: enhanced desire to impress, peers introducing a “cognitive load”, the capacity for peers to shift orientation toward reward, or heightened physiological and emotional arousal in the context of peer evaluation [308].

There is substantial evidence that some alleles in the dopaminergic system (such as those for *COMT*, *DAT1*, *MAOA*, and genes for dopamine receptors, especially *DRD4* and *DRD2*) and the serotonin-transporter-linked polymorphic region (*5-HTTLPR*) gene variants are related to executive attention, temperament, attachment, psychosis risk, and sensation seeking [309,310]. One of these genes, the gene for the dopamine receptor 4 (*DRD4*) in chromosome 11, was found to influence sensation-seeking behavior as early as 18–20 months in interaction with the quality of parenting [311]: when the 7-repeat allele was present, relatively low-quality parenting produced higher sensation-seeking ratings, but when the 7-repeat allele was absent, sensation seeking was moderate and low, regardless of parenting quality. This finding of the susceptibility of children and adults with the 7-repeat allele to parental and other environmental influences has been replicated many times [312,313,314], supporting the view that reward processing in appetitive motivation has an important role in sensation seeking. Besides sensation seeking in toddlers when combined with poor parenting, *DRD4* gene polymorphisms have been associated with several other phenotypes, including an increased risk of attention deficit hyperactivity disorder (ADHD), impulsivity, and lower levels of response inhibition [311,315].

On a number of occasions, patient S. M. reported a high level of excitement and enthusiasm while riding a rollercoaster and also wanted to try skydiving [316]. While these observations suggest a high level of “sensation seeking”, in everyday life S.M. rarely engaged in purposeful risk-taking behavior, perhaps due to her inability to afford such activities [316].

Altogether, these results suggest that damage of the amygdala causes behavioral disinhibition that may interact with unemotional traits in a number of ways. Low levels of fear may result in unresponsiveness to parental discipline, ambivalence about parental or peer disproval, and low levels of anxiety in response to one’s own misbehavior [317]. These factors conceivably combine to produce a child who is unafraid of being disciplined, unmotivated to behave appropriately, and unable to feel remorse for his or her misbehavior. Therefore, disinhibition may represent a risk factor for reactive aggression as well as for sensation seeking and a lack of empathy and remorse. Reactive aggression and psychopathology both implicate hypoactivity of both the amygdala and OFC [318,319].

## 13. Decision-Making and Interdependence of Emotion and Cognition

The ability to anticipate the outcome, as well as the time available for reaching a decision, play important roles in decision-making, too. Only humans and some non-human primates, and perhaps some other species (elephants, dolphins), can be surprised when events do not unfold as expected [320]. Surprise, one of the primary emotions, is the reflection of the uncertainty of the outcome and the connection between cognition and affect since it simultaneously involves probability estimation, intuition and the expected reward, and, depending on the outcome, secondary emotions of sadness or rejoicing arise. The pattern of brain activity during surprise mostly includes the inferior frontal gyrus of the right hemisphere, followed by the ventrolateral OFC and the attention-related areas of the cingulate cortex and precuneus. As the response time is faster, the more emotionally charged the stimulus is, emotions accelerate the resolution of such conflicts and reduce the time for which the individual is unable to (re)act [321]. As stated earlier, such monitoring for possible conflicts and an intuitive system of emotional go/no-go decisions are mediated by neurons of the ACC, whose main feature is the high speed of decision-making since they do not search for the best possible answer, but only the emotional dimension of a better immediate answer that gives a higher probability of survival. 

The belief that reason and feelings are separate systems has a long history in Western philosophy, literature, and science. However, cognition and emotion are today understood as interrelated phenomena, and their integrated action is necessary for normal adaptive functioning [322,323]. Neural circuits underlying emotions and cognition are in constant interaction with one another and, as such, they affect attention and perception, decision-making and reasoning in a complementary fashion [324]. It is a reciprocal relationship, hence emotional states can strongly influence selective attention, working memory, and cognitive control; similarly, attention and working memory contribute to the voluntary regulation of emotions [325,326].

The findings from human studies are consistent with those obtained from animals. For example, studies using fMRI have shown increased levels of activity in the amygdala in response to a neutral stimulus paired with an aversive event compared to a neutral stimulus that did not anticipate an aversive event [327]. Moreover, finding that patients with damage to the amygdala do not show a conditioned, autonomic response to visual or auditory stimuli is also consistent with the results of animal studies [328]. The amygdala is involved in the development of phobias as well as in the maintenance of specific fears and generalized anxiety, together with the vmPFC [329,330,331].

Studies of the effects of emotions on attention have shown that emotionally charged stimuli are more likely to reach consciousness in situations where attention capacities are limited and that the amygdala plays a key role in mediating that effect [332,333]. Generally, the effect of emotions on memory is two-fold: in certain conditions, emotions improve memory, and in others they interfere with it, depending on the networks used. Using experimental tasks of working and episodic memory with simultaneous imaging of brain activity, the effect of amygdala activation results in emotional distractors having a short-term negative effect on working memory, and a long-term positive effect on improving episodic memory through increased activity of the amygdala and hippocampus in combination with decreased activity of the dlPFC, combined with increased activity of the ventrolateral PFC (vlPFC) [324,334]. Moreover, people that are more sensitive to the disruptive effects of emotions on working memory showed a higher degree of amygdala activity and a lower activity of PFC. A better understanding of the mechanisms that mediate the different effects of emotions on cognition is definitely a way to understand affective disorders, such as anxiety and depression, since in these disorders this interaction is dysfunctional. As the amygdala participates in the consolidation of fear-related memories, its dysfunction is thought to either lower or raise the threshold for activation in anxious situations. If it becomes too low, hyperactive anxiety states and phobias can occur during negative conditioning or learning aversive reactions. Individual differences in the volume and concentration of the gray matter of the amygdala may also underlie the association between personality traits, especially extraversion and neuroticism. For example, one study showed that extraversion is positively correlated with the concentration of gray matter in left amygdala, while neuroticism is negatively correlated with gray matter concentration in the right amygdala [330,335].

The lateral prefrontal cortex (lPFC) is considered a major area of integration of emotion and cognition [336,337,338,339,340]. However, it is not a single area of the brain that has this supervisory role, but instead a series of dynamically interconnected neural networks of which the central places are occupied by hubs of connections that are critical for the regulation of information flow and the integration of information between the dlPFC, vlPFC, OFC, vmPFC, ACC, cerebral cortex of the intraparietal sulcus, anterior insula, and amygdala [335,339]. In addition, the anterior insula critically limits the capacity of the cognitive control network to mediate the coordination of thoughts, feelings and actions [340]. Emotions can be understood only in the context of adaptive, synchronized interactions of widely distributed cortical and subcortical neural networks that mediate complex adaptive behaviors, such as perception, cognition, motivation, and actions in which the amygdala plays a central modulatory role [339,341,342,343]. Human intelligence arises from the complex interaction of cognitive processes that are modified by different levels of emotional self-awareness and motivation. Awareness of one’s emotions and feelings and the ability to empathize and use judgment are required abilities and skills to enable cognitive embodiment, social awareness and self-regulation of cognitive processes.

## Figures and Tables

**Table 1:** Division and characteristics of the two main types of aggression, and the role of the amygdala. ANS—autonomic nervous system; OFC—orbitofrontal cortex; PAG—periaqueductal gray matter; PTSD—post-traumatic stress disorder; TBI—traumatic brain injury; vmPFC—ventromedial prefrontal cortex. Information according to Blair (2010) [215], Begić (2014) [216], Bogerts et al., (2018) [217], Farah et al., (2018) [218], and Gouveia et al., (2019) [138].

Aggression Type	Characteristics	Conditions in which It Occurs	The Role of the Amygdala
Impulsive (reactive)	Unplanned, caused by increased arousal to a provocation or a threat, accompanied by a feeling of anger; primary intention is to destroy the victim (usually the provocateur)	Intermittent explosive disorder, autism, impulsive type of emotionally unstable personality, post-TBI disorders, PTSD	Increased activity, especially of the amygdala in the right hemisphere, with decreased control of the amygdala via PFC (decreased PFC activity); increased activity of the ANS, which includes increased reactivity of the “threat system” (medial part of the amygdala, hypothalamus, PAG)
Planned (proactive, instrumental)	Planned in advance, associated with a reduced degree of compassion (empathy); intention is to achieve a certain goal (usually some personal benefit)	Antisocial (DSM5)/dissocial (ICD-10) personality disorder	Decreased volume of amygdala and its activity, especially in tasks involving compassion; decreased amygdala functional connectivity with vmPFC, OFC, and posterior cingulate cerebral cortex, decreased OFC activation to provocation

**Table 2:** Selected clinical cases of disturbed emotional experience and behavior. See text for details.

Case	Basic Neuropathological Findings	Altered Behavior	Reference(s) No.
Phineas Gage	Bilateral damage of the frontal lobe, especially vmPFC, including the extensive damage to the white matter of the frontal lobe as well as the anterior parts of temporal lobe and amygdala (amygdala disconnected from the frontal lobes)	Careful and reliable person before the injury after the injury became emotionally unstable, impulsive, unpredictable, dishonest, capricious, reckless, having disturbed social skills and difficulties in making decisions (“no longer Gage”)	[270,271,272,273]
Patient S.M.	Bilateral calcification of the amygdala and periamygdaloid gyrus due to the Urbach–Wiethe disease	Patient S.M. had highly specialized impairment associated with the emotion of fear: she could not experience fear nor she could recognize facial expressions showing fear	[61,274,275]
Boy B.W.	Congenital ventromedial prefrontal cortex malformation involving Brodmann areas 11, 12, 25 and 32, clusters of dysplastic neurons in the left amygdaloid nucleus	Throughout his childhood, this boy with a relatively normal cognitive performance on standard neurophychological tests displayed incremental emotional instability, impulsivity, lack of empathy, hypersexuality, and had been manipulative and aggressive towards others, including his own parents	[276]
Patient B.	Bilateral destruction mainly of the insula due to *Herpes simplex* infection, but to a lesser extent also of the orbitofrontal and temporal cortex, anterior part of the ACC, hippocampus, EC, amygdala and a part of basal telencephalon	Severe global amnesia, dense impairment of retrograde memory and shallow mental content, but, except for taste and olfaction, all aspects of feeling were intact	[183]
Patient Roger	Bilateral damage to insula, ACC, and amygdala due to *Herpes simplex* infection	Major deficits included global amnesia, anosmia (the inability to percieve smell/odor), and ageusia (the inability to taste), while his experience of pain was intact, at times even excessive	[184]
Patient A.P.	Selective bilateral damage to the amygdala due to the Urbach–Wiethe disease	A pleasant, cheerful young woman notable for her tendency to be somewhat coquetting and disinhibited, e.g., she had been quick to become friendly with examiners, and had often made mildly innapropriate sexual remarks. She had also suffered from a significant defect in visual, nonverbal memory, executive control manifesting with innapropriate social behaviors, and had deficits on tests of category formation, cognitive flexibility, and abstract reasoning	[277,278]

## Abbreviations

A (AMY)—amygdala
5-HT—5-hydroxytryptamine (serotonin)
ACC—anterior cingulate cortex
AMPAR—α-amino-3-hydroxy-5-methyl-4-isoxazolepropionic acid receptors
ANS—autonomic nervous system
AP-1—transcription factor activating protein 1
ATP/Ado—adenosine triphosphate/adenosine
BA—Brodmann’s area
BF—basal forebrain
BLA—basolateral nucleus of amygdala
BNST—bed nucleus of stria terminalis
BPD—borderline personality disorder
CE—central nucleus of amygdala
CN—caudate nucleus
CNS—central nervous system
Co—cortical nucleus of amygdala
CPRN—caudal pontine reticular nucleus
CRH/CRF—corticotropin releasing hormone/factor
CS—conditioned stimulus
DA—dopamine
dlPFC—dorsolateral prefrontal cortex
dmPFC—dorsomedial prefrontal cortex
DRN—dorsal raphe nucleus
DSM-5—Diagnostic and Statistica Manual of mental disorders, 5^th^ revision
DTN—dorsal tegmental nucleus
EC—entorhinal cortex
EEG—electroencephalogram
FLAIR—fluid attenuated inversion recovery MRI sequence
fMRI—functional magnetic resonance imaging
GABA—gamma (γ) aminobutyric acid
GAD—generalized anxiety disorder
H—hippocampus
HF—hippocampal formation
IN—intercalated neurons of the amygdala
ICD-10—International Classification of Diseases, 10th revision
LA—lateral nucleus of amygdala
LC—locus coeruleus
LH—lateral hypothalamus
lPFC—lateral prefrontal cortex
LTD—long-term depression
LTP—long-term potentiation
MDD—major depressive disorder
MDMA—3,4-methylenedioxymethamphetamine (ecstasy)
ME—medial nucleus of amygdala
MGN—medial geniculate nucleus of thalamus
mPFC—medial prefrontal cortex
NAc—nucleus accumbens septi
NAc MNS—medium spiny neurons of NAc
N. V—trigeminal nerve
N. VII—facial nerve
NMDAR—*N*-methyl-D-aspartate receptors
OFC—orbitofrontal cortex
OXT—oxytocin
P—putamen
PAG—periaqueductal gray
PBN—parabrachial nuclei
PCC—posterior cingulate cortex
PL—paralaminar nucleus
PNS—peripheral nervous system
PTSD—post-traumatic stress disorder
PVN—periventricular nucleus
rmPFC—rostromedial prefrontal cortex
RMTg—rostromedial tegmental nucleus
BDNF—brain-derived neurotrophic factor
DTI—diffusion tensor imaging
SNc—substantia nigra, pars compacta
TBI—traumatic brain injury
UC—unconditioned stimulus
vlPFC—ventrolateral prefrontal cortex
vmPFC—ventromedial prefrontal cortex
VPL—ventroposterolateral nucleus of thalamus
VPM—ventroposteromedial nucleus of thalamus
VTA—ventral tegmental area
